# Chemosensitizer Loaded NIR-Responsive Nanostructured Lipid Carriers: A Tool for Drug-Resistant Breast Cancer Synergistic Therapy

**DOI:** 10.1021/acsabm.4c01675

**Published:** 2025-02-18

**Authors:** Cigdemnaz Ersoz Okuyucu, Gokce Dicle Kalaycioglu, Ayse Kevser Ozden, Nihal Aydogan

**Affiliations:** 1 Department of Chemical Engineering, Hacettepe University, Beytepe, Ankara 06800, Turkey; 2 Faculty of Medicine, Medical Biology Department, Lokman Hekim University, Ankara 06530, Turkey

**Keywords:** nanostructured lipid carriers, photothermal therapy, NIR-triggered release, multidrug resistance, doxorubicin, breast cancer, verapamil

## Abstract

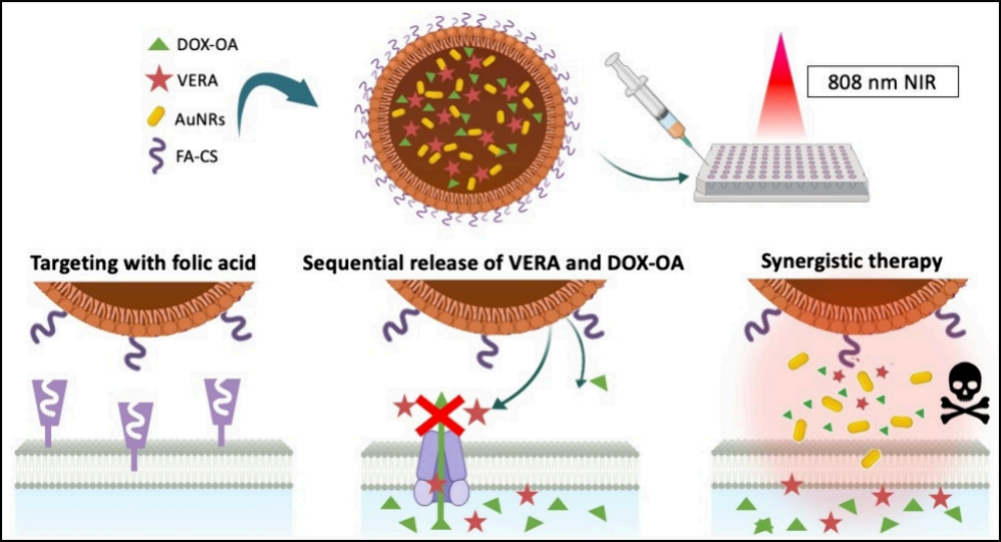

Although numerous technical advances have been made in cancer treatment, chemotherapy is still a viable treatment option. However, it is more effective when used in combination with photothermal therapy for resistant breast cancer cells. This study introduces a smart drug delivery system, (DOX-OA+VERA+AuNRs)@NLC, which is designed for dual chemo/photothermal therapy of multiple-drug-resistant breast cancer. Type-III nanostructured lipid carriers (NLCs) were used as drug delivery systems, where nano-in-nano structures offer several advantages. Doxorubicin (DOX) was used as the antitumor agent by ion-pairing it with oleic acid (OA) to increase the DOX loading capacity, as well as to reduce the burst release of the drug. Verapamil (VERA), which was used as a chemosensitizer to overcome the multiple-drug resistance, was co-loaded with DOX-OA. Gold nanorods (AuNRs) were exploited as the photothermal therapy agent in photothermal therapy (PTT) application, which would have a synergistic relation with chemotherapy. The release of DOX-OA and VERA from NLCs was studied *in vitro* by triggering with NIR laser irradiation. Thus, an all-in-one drug delivery system was designed to release the active pharmaceutical ingredients (APIs) at higher concentrations in the desired region and provide both chemo/PTT. Besides, the application of a folic acid-chitosan (FA-CS) coating to NLCs has facilitated the development of systems capable of targeting and specifically releasing their cargo within cancerous tissues while preserving their surrounding environment.

## Introduction

Breast cancer is currently one of the lethal diseases affecting many people worldwide.^1^ This cancer has the highest number of cases and deaths compared to all other cancers in women.^2^ As a prominent treatment, chemotherapy has been applied over the years, even though the treatment of breast cancer depends on the particular biological conditions of the tumor.^3,4^ Although chemotherapy is commonly used, it often faces challenges due to multidrug resistance (MDR). MDR is a phenomenon of cancerous cell resistance to APIs.^5,6^ Preventing MDR is a major challenge in the treatment of breast cancer in the clinic and the laboratory.^7,8^ Generally, MDR can be caused by P-gp overexpression.^9^ Hence, only chemotherapy cannot be efficient even if higher-dose APIs are reached by the resistant cell line. Preventing MDR has been an important challenge for the treatment of breast cancer.^10^

Although the use of multiple APIs with varying therapeutic outcomes either at the same time or in a specified order can help overcome MDR, a potential drawback is the increased risk of drug–drug interactions and potential adverse effects.^11,12^ For this reason, the dual use of a chemotherapeutic agent and a chemosensitizer drug that tends to reduce MDR would be more beneficial in treatment by increasing the efficacy of the chemotherapeutic agent. DOX hydrochloride is an anthracycline antibiotic and also an active antitumoral agent that is used in the treatment of many cancers, breast cancer being one of them.^13,14^ The drug efflux pump effect of DOX can be prevented by increasing its concentration; however, this would result in significant toxicity.^15^ Yet, during chemotherapy, APIs may spread to undesired areas in the bloodstream and, worse, lead to systemic accumulation. Hence, a chemosensitizer would eliminate this challenge.^16^ VERA hydrochloride is one of the good options due to it blocking the drug efflux pump, which is a chemosensitizer agent and P-glycoprotein (P-gp) inhibitor that can reverse P-gp-associated MDR with a combined usage of DOX.^17,18^

In recent years, it has become popular to widely use drug delivery systems to enhance the bioavailability, biodegradability, and sustained release of APIs.^19−21^ Lipid-based systems like NLCs are frequently desired as they do not have toxic effects on the human body.^22,23^ NLCs build in liquid and solid lipids at the same time in their defective crystalline structure, and these imperfections may provide nanocompartments for drug loading, especially for hydrophobic-based APIs.^24^ NLCs that possess a nanocompartment within their structure are referred to as multiple-type (type-III) NLCs.^25^ These NLCs have gained attention for their ability to enhance drug loading capacity and enable a more sustained release. This is achieved by exploiting the lower solubility of the drug in solid lipids compared to liquid lipids.^25,26^ Consequently, the drug dissolves preferentially in the liquid lipid compartment, while the outer solid lipid matrix aids in achieving prolonged drug release.

Multi-type NLCs have a comparatively high capacity for encapsulating hydrophobic drugs.^27^ Yet, DOX and VERA's solubility in water is around 10 and 83 mg/mL, respectively. Enhancing the drug molecule’s hydrophobicity by making an ion pair with a more lyophilic molecule would be a solution to come through this issue.^28^ In literature, there are some studies that have ion-paired DOX with OA.^29,30^ The OA ion pair DOX (DOX-OA) is stable in a neutral medium (pH 7.4) such as blood, but unstable in an acidic pH such as tumor tissues.^30^

The release of APIs from drug delivery systems (DDSs) through stimuli changes such as temperature has been reported in the literature.^31−33^ Zhang et al. reported that liposomes exhibit structural perturbations over 42 °C because of lipid phase liquefaction.^34^ The characteristics of lipid-based DDS make them suitable as temperature-responsive DDSs at target sites. AuNPs have been used in the literature widely due to their characteristic to convert optical light into heat when irradiated with 530–700 nm light, demonstrating their photothermal properties.^35^ Light with wavelengths over 650 nm has relatively low absorption and scattering in normal tissues, allowing it to penetrate deeply without damaging healthy cells.^36^ A photothermal agent can readily harness this optical energy to convert it into heat energy, culminating in its accumulation within the tumor. These photothermal agents can be investigated for their potential in combined PTT with conventional chemotherapy or photodynamic therapy, presenting a promising avenue toward the efficient treatment of cancer. Tumor cells have more high-temperature sensitivity than healthy cells and show an accelerated death rate under the hyperthermia.^37,38^ Thakur and co-workers reported in their study that the local hyperthermia provided by AuNPs, besides killing cancer cells by the increasing of temperature, could cause a phase transition of the cancerous cell membrane that enhances the membrane fluidity and permeability of the cells, thus enhancing the drug uptake and further improves the effect of the DDS.^35^ Thus, drug and AuNP-encapsulated nanocarriers would provide a highly developed platform to achieve the synergistic effect of chemotherapy and PTT. In order to benefit from this combined approach, an active targeting can be more beneficial in terms of increasing the effectiveness of the treatment. For that purpose, surface modification of the DDS is one of the used approaches. Targeting the folate receptors could be a reasonable way since aggressive and metastatic triple-negative breast cancer cells such as MDA-MB-231 are enriched with the folate receptor.^39^ In the present study, a lipid-based smart carrier was obtained by encapsulating the dual drugs DOX-OA and VERA in NLCs. After that, the dual drugs and AuNRs were loaded into the optimal NLC structure, and cumulative release was performed at pH 5.5 and 7.4. The aim was to enhance the effectiveness of the chemotherapeutic agent by achieving a higher release of VERA compared to DOX-OA. AuNRs have an optimum size that has been shown to be effective as a PTT agent to kill cancer cells as well as to control the amount of drug released. NLCs were also decorated with FA-conjugated CS for targeting. The resulting effects were investigated using the MDA-MB-231 cell line, a triple-negative cell line that is one of the most difficult cancer cells to target and treat. Moreover, these cells were also treated for them to develop a resistance to the chemotherapy agent. In light of these results, it can be concluded that this functionalized, smart, and state-of-the-art DDS will play a crucial role in treating breast cancer, even in the presence of MDR, where combined chemotherapy and PTT are used.

## Experimental Methods

### Materials

Gold(III) chloride trihydrate, sodium phosphate dibasic dodecahydrate, Pluronic F-127, sodium phosphate monobasic monohydrate, sodium chloride, FA, chloroform, and methanol were purchased from Sigma-Aldrich (St. Louis, MO, USA). NaBH_4_, which was used as a reducing agent during gold synthesis; Tween-20; CTAB; and stearic acid were obtained from Merck (Darmstadt, Germany). AgNO_3_ and *tert*-butylammonium bromide were obtained from Acros Organics (Carlsbad, CA, USA). DOX hydrochloride and VERA hydrochloride were obtained from Toronto Research Chemicals (North York, Canada). Sodium bicarbonate and OA were purchased from Fisher Chemicals (Loughborough, UK). For all experiments, ultrapure (UP) water, having a resistivity of 18.2 MΩ cm, was obtained using a Millipore Direct-Q3 UV water purification system. All chemicals were utilized as received without additional purification.

### Preparation of (DOX-OA+VERA+AuNRs)@NLC

NLCs were synthesized as a DDS using the melt-emulsification method, as specified in the previous study.^40^ To summarize, a combination of stearic acid and OA weighing 95 mg was blended and included in 30 mL of UP water and 98.4 mg of Pluronic F-127 once both solutions attained a temperature of 75 °C. Here, stearic acid was used as a solid and OA was used as a liquid lipid. Also, Pluronic F-127 was used to reduce the interfacial tension between the lipid matrix and the lipid nanoparticle water phase. Additionally, acetone and ethanol were added to the solution, respectively, before being added to the water phase. Subsequently, this final emulsion was allowed to freeze at −20 °C, up to at least 3 h.

AuNRs were synthesized by using the seed-growth method. The method for synthesizing AuNRs is a slightly modified version of Liu and his co-workers’ procedure.^41^ In brief, 0.25 mL of a 10 mM HAuCl_4_ solution and 10 mL of a 0.1 M CTAB solution were mixed at 30 °C. Subsequently, a 10 mM (0.60 mL) NaBH_4_ solution was added to the mixture under vigorous stirring. At this point, the NaBH_4_ solution was kept ice cold. In order to decompose excess NaBH_4_, the seed solution underwent stirring for 5 min. Starting the silver(I)-assisted growth portion required the mixture of 0.5 mL of HAuCl_4_ (10 mM) and 0.1 mL of AgNO_3_ (10 mM) with 10 mL of CTAB solution (0.1 M). By adding AgNO_3_ to the growth solution, the aspect ratio can be easily controlled by the silver concentration, thereby enabling the production of AuNRs with high yield.^42^ Next, 0.2 mL (1.0 M) of HCl was added, reducing the pH to 3–4 to ensure structural stability. After that, in order to reduce the gold from Au(III) to Au(I), 0.08 mL (0.1 M) l-ascorbic acid was added into the mixture. In the last stage, 24 μL of seed solution was introduced to the growth solution, and under gentle stirring for 2 h, the solution was retained at 30 °C. The aqueous solution of AuNRs was centrifuged at 10,000 rpm for 30 min to remove excess and/or free CTAB molecules.

The DOX-OA ion pair was prepared with several modifications to the procedure reported by Zhao et al.^30^ Briefly, the DOX hydrochloride (5 mg/mL) and sodium bicarbonate solutions (50 mg/mL) were mixed by magnetic stirring for 10 min. Then, a solution of OA (50 mg/mL) in ethanol was added to the mixture. After stirring continuously for 90 min, the mixture was centrifuged at 5000 rpm for 30 min. The precipitate was washed three times with distilled water under the same conditions to remove excess OA.

Preparation of (DOX-OA+VERA+AuNRs)@NLC (in the NLC preparation procedure mentioned above) requires specific amounts of DOX-OA, VERA, and AuNRs additions to the oil phase mixture composed of stearic acid and OA. After that, the oil phase, which was loaded with the photothermal agent and drugs, was blended with the water phase. Subsequently, the two-phase mixture was prepared in a manner analogous to the aforementioned NLC preparation during the loading of the APIs into the NLC, and two ratios were utilized, 1:15 and 1:144 (drug:lipid, w/w%). The concentration of DOX-OA to VERA remained constant at 1:1.5 (DOX-OA: VERA, w/w%).

### Characterization of (DOX-OA+VERA+AuNRs)@NLC

Hydrodynamic diameters of NLC, AuNR-loaded NLCs (AuNR NLC), and API-loaded NLCs (DOX+VERA NLCs) were measured by dynamic light scattering (DLS, ALV-CGS-3 Compact Goniometer). The samples were prepared by diluting them in 1:100 ratio with DI water for the DLS measurements. Samples were analyzed at a 90° angle, with at least six repetitions. Atomic force microscopy (AFM, psia Corporation, XE-100E) was also used to determine the particle’s morphology and size. AFM characterizations were performed after the diluted samples were dropped on a microscope slide and allowed to dry.

### Differential Scanning Calorimetry Measurements

Differential scanning calorimetry (DSC, PerkinElmer PYRIS Diamond) analysis was performed to investigate the crystallinity of NLCs and the effect of encapsulated drugs and NPs. After the samples were lyophilized, they were placed in the aluminum pan, and the measurements were carried out at a rate of 10 °C/min under a nitrogen gas environment in the range 10–250 °C. The crystallinity indexes of the particles were calculated according to the formula below:
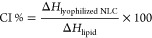
1Here, CI% represents the percentage of crystallinity index, and Δ*H* represents the enthalpy change.^43^

### Encapsulation Efficiency (EE%) and Drug Loading (DL%)

In order to assess the amount of nonencapsulated DOX-OA and VERA in the NLC structures, NLC dispersion was centrifuged at 8000 rpm for 15 min. The absorbance values of the supernatants were measured at 279 nm for VERA and 485 nm for DOX-OA by a UV–vis spectrophotometer (UV–vis, Thermo Scientific, GENESYS 10S). The concentration (*c*) of DOX-OA and VERA was obtained based on the standard curve: *A* = 25.005*c* (mg/mL) for DOX-OA and *A* = 9.6811*c* (mg/mL) for VERA, where *A* is the absorbance. The EE% and DL% were calculated using eq 2 and eq 3, respectively:^44^
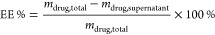
2
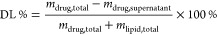
3where *m*_drug,total_, *m*_drug,supernatant_, and *m*_lipid,total_ are the total amount of drug, the free amount of drug in the supernatant, and the total amount of lipid, respectively.

### *In Vitro* DOX-OA, VERA Release, and NIR-Triggered Release

DOX from NLCs was separately investigated in the presence of both AuNRs and only APIs. Drug release experiments were conducted in 50 mL of PBS solution containing Tween-20 (0.7% (w/v%)) at 37 °C. A 1 mL portion of drug-loaded NLC dispersion (0.32 mg drugs/mL) was placed into the dialysis tubing cellulose membrane bag (typical molecular weight cut-off = 14 kDa). Then, to ensure sink conditions were maintained, the reaction was added to the release medium. The absorbance values of the drug molecules diffusing from the dialysis bag through the membrane into the medium were noted within specific periods of time, and releases were observed for 48 h periods. While pH 7.4 is the normal pH of blood and most bodily fluids, the pH of the invasive MDA-MB-231 breast cancer cells can decrease up to pH 5.5.^45^ To simulate the pH levels of blood and cancer cells, we conducted experiments at pH 7.4 and pH 5.5, respectively.

NIR laser (808 nm, 2.5 W, PSU-H-LED laser power supply, Changchun New Industries Optoelectronics Technology) was used to understand the photothermal effect of the multimodal treatment. For NIR-triggered release experiments, NIR laser irradiation was applied on a dialysis bag at 808 nm, 2.3 W for 5 min. A laser power supply was placed 10 cm from the dialysis bag. The temperature was recorded with a thermal camera (FLIR E54) to understand whether the concentration increase was related to the temperature increase. After each heating–cooling cycle, the absorbance of DOX-OA and VERA was recorded via UV–vis spectrophotometer, similar to NIR-off release studies. All experiments were repeated at least three times to ensure accuracy.

### Drug Release Kinetic Models

In order to understand the release kinetics, Zero order, First order, Higuchi, Korsmeyer-Peppas, and Hixson-Crowell models, which are frequently used in the literature, were examined. The linearization method (*R*^2^) was used to understand which kinetic model fits the release. The “*n*” value for the Korsmeyer-Peppas model has also been calculated. The difference in the release kinetics was observed at different pH values and in the presence of AuNRs.

### Preparation of FA-CS

A mixture of 1-(3-(dimethylamino)propyl)-3-ethylcarbodiimide hydrochloride (EDC) and FA (0.065M) in anhydrous DMSO was prepared. The 1% (w/v) CS solution was stirred for 4 h in acetate buffer, while the FA solution was prepared by stirring for 2 h in anhydrous DMSO with a 1:1 molar ratio of EDC in a nitrogen atmosphere. During the initiation of the reaction, the CS solution was added dropwise to the FA solution. The reaction, lasting for 16 h in the dark, was terminated by adjusting the pH of the medium to 9 with 1 M NaOH. The bright yellow precipitates observed at this stage were collected by centrifugation at 2500 rpm for 5 min. To separate the obtained product from the unreacted portion, the solid was redispersed in PBS and dialyzed against PBS for 2 days. For the removal of PBS salts, the sample was further dialyzed in pure water for the next 2 days. Upon completion of dialysis, the product was dried under a vacuum.

Also, in the stage of coating the surface of NLCs with FA-CS, an aqueous solution whose pH was adjusted to 9 using acetic acid (2%) and NaOH (1 M) solutions was used.

After the determination of the FA-CS conjugate using FTIR, UV–vis spectrophotometry was used to understand how much FA was bound to the amine groups in the CS chain. FA/NH_2_ ratio was calculated as 0.084.

### Cell Culture Studies

Cytotoxicity studies on NLCs were carried out by using the MTT (3-(4,5-dimethylthioazol-2-yl)-2,5-diphenyltetrazolium bromide) method with the L-929 fibroblast cell line. Absorbance values were measured in an ELISA reader (Coulter, USA) at 440 nm at the end of the incubation. Percent viabilities were determined based on the reference wells. Cell culture studies with cancerous cells were conducted using a highly metastatic breast cancer cell line, MDA-MB-231, purchased from the American Type Culture Collection (ATCC, Wesel, Germany). MDA-MB-231 cells were counted and seeded in 96-well cell culture dishes (5000 cells/well) at 37 °C for 24 h in 5% CO_2_. MDR-resistant cells, specifically MDA-MB-231^R^, were obtained by culturing cells in gradually increasing amounts of DOX (5 nM to 200 nM). Surviving cells gained resistance after each treatment step and were incubated in their nutrient medium under a 5% CO_2_ environment at 37 °C.

To determine the influence of hyperthermia effect on the viability of MDA-MB-231^R^ cells, a cell suspension was illuminated with an 808 nm NIR laser irradiation for 5 min at 2.3 W. The increase in temperature caused by laser exposure within a controlled environment was monitored using a thermal camera.

### Lateral Motility Assay

To evaluate the metastatic potential of the highly metastatic MDA-MB-231 cells post-treatment, their lateral motility was assessed using a wound-healing assay. The cells (2 × 10^5^ cells per well) were preferred to seed in six-well plates (Nest Scientific USA Inc.). Once the cells reached confluency, three wounds were created in each well using a pipette tip. Wound widths were recorded under an inverted microscope (Leica, Wetzlar, Germany) after the wells were rinsed with a fresh medium. Cells were treated with DDS free, DOX-OA+VERA NLC, (DOX-OA+VERA+AuNRs)@NLC, and (DOX-OA+VERA+AuNRs)@NLC-FA-CS for 48 h. The NIH ImageJ program was used to measure wound areas on pictures at 0 and 48 h.

### Statistical Analysis

Data were presented as mean ± standard deviation (SD), and all experiments were performed at least three times. Statistical analysis was conducted using one-way ANOVA with Tukey’s multiple-comparisons post-test comparing all conditions. *p* values of ≤0.05 were considered statistically significant.

## Results and Discussion

### Preparation and Characterization of (DOX-OA+VERA+AuNRs)@NLC

Since the AuNRs and several drugs were intended to be encapsulated in NLCs, the work was started with the preparation of the NLCs. NLCs with varying percentages of OA (liquid lipid) were analyzed by DLS and AFM (Table S1). The results indicated that there was a decrease in particle size with increasing amounts of OA up to 30%, although it increased when the amount of OA reached 40%. The DLS measurements showed that NLCs with 20, 30, and 40% OA had particle sizes of 105.84 ± 40.57, 72.02 ± 31.39, and 205.40 ± 79.64 nm, respectively (Figure S1.B,D,F). Similarly, the AFM measurements showed that the particle sizes for NLCs with 20, 30, and 40% OA were 135.20 ± 48.48, 66.35 ± 14.85, and 109.02 ± 32.19 nm, respectively (Figure S1.A,C,E). Since the DLS measurements were conducted on liquid samples, the hydrodynamic diameter influenced the particle size. The sizes were anticipated to be larger when the samples were examined in the solid state.

Upon examination of the size distributions in detail, it was found that some of the particles in the 20% NLCs were agglomerated, as was evident from both DLS and AFM measurements. However, 30% OA-containing NLCs did not show any agglomeration, as confirmed by both characterization methods. The reason for the decrease in particle sizes up to 30% OA was the reduction in surface tension caused by the increase in OA, resulting in smaller NLCs. Higher OA content decreased the viscosity in the NLCs and consequently decreased the surface tension, leading to the formation of smaller particles with smoother surfaces.^46^ However, beyond 40% OA, the particles began to agglomerate again. Therefore, using NLCs with 30% OA is advantageous for particle size control.

**Figure 1:**
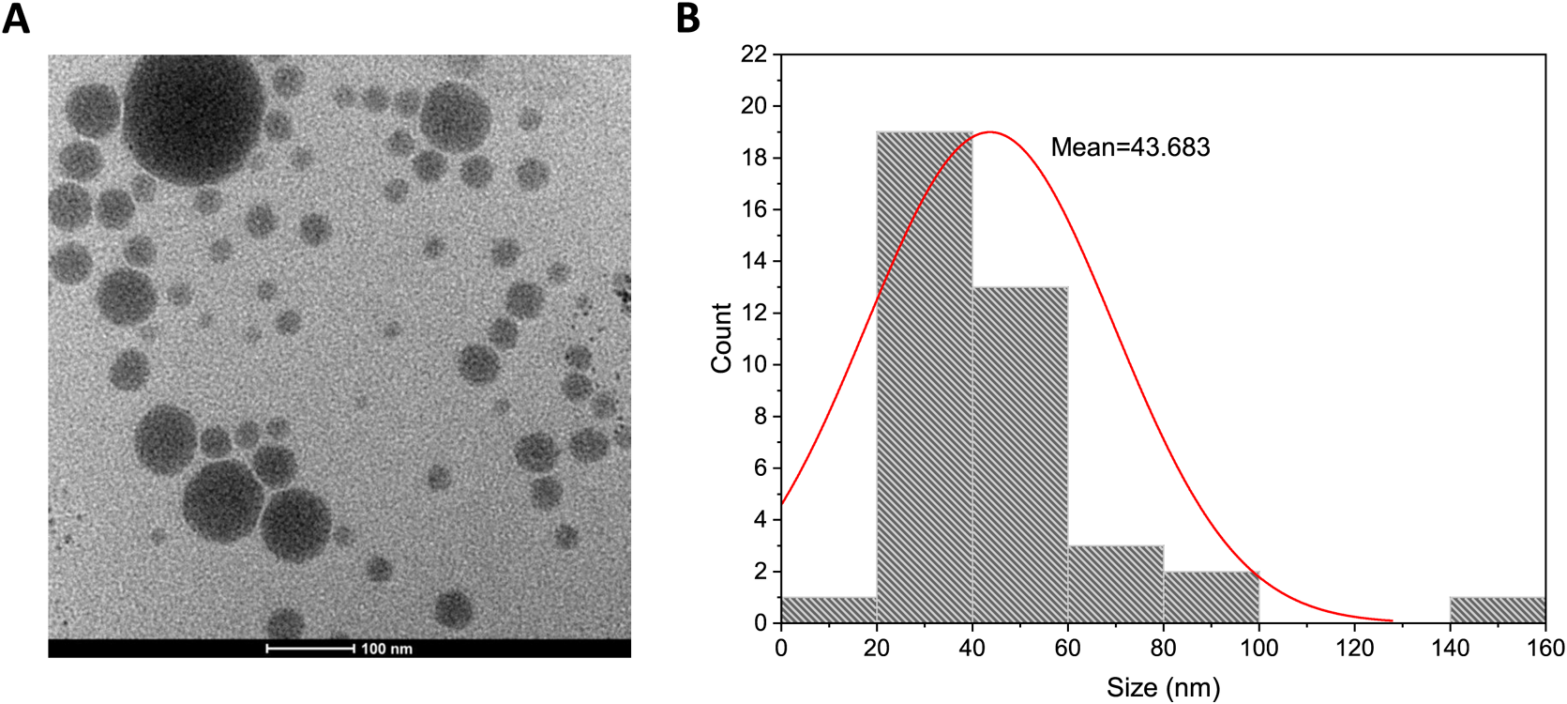
(A) Bright-field TEM image of NLC containing 30% OA, and (B) size distribution of NLC with 30% OA, derived from the Bright-field TEM image. Data are presented as mean ± SD (*n* = 3).

TEM analysis was conducted to complement the AFM and DLS size measurements and to provide insight into the geometry of the particles. Figure 1A presents the TEM image of NLCs with 30% OA content. The particles exhibit a spherical morphology consistent with the AFM results. Analysis of the size distribution revealed an average particle size of 43.69 ± 16.14 nm (Figure 1B). The size of NLCs was calculated to be 72.02 ± 31.39 and 66.35 ± 14.85 nm from DLS and AFM, respectively. Given that the hydrodynamic radius can be measured using DLS, it makes sense to give the largest size. Based on the TEM analysis result, there are several benefits to the small size of NLCs. In the continuation of the study, it is expected that the drugs and AuNRs that are planned to be encapsulated in NLC will be enhanced in size. Small particle sizes are crucial for intravascular or intravenous administration. For these reasons, the smallest NLCs were chosen for further studies. Also, a short-term stability test was conducted to evaluate the stability of the NLCs. AFM analysis revealed that the size of NLCs increased by 3.22% after 4 days at −20 °C. Additionally, the zeta potential was measured at −30 mV, indicating good colloidal stability.

DOX-OA and VERA active ingredients were loaded into NLC in varying drug/lipid combinations. DLS and AFM were used to examine the change in the size of these drug-loaded NLCs. When Table 1 was examined for both ratios (1:15 and 1:144 (drug:lipid)), the size of the drug-loaded NLCs increased in comparison to the nonloaded NLCs, which have a diameter of 43.69 ± 16.14 nm. The ratio of 1:144 (drug:lipid), as the lowest concentration value that can be calculated with laboratory devices, was chosen to examine the properties of low-concentration APIs encapsulated in NLCs. At a ratio of 1:144 (drug/lipid), the size of NLCs encapsulated with DOX-OA, VERA, and DOX-OA+VERA was calculated by AFM to be 204.5 ± 32.75, 170.0 ± 29.96, and 320.8 ± 57.63 nm, respectively. In addition, the size of NLCs loaded with DOX-OA, VERA, and DOX-OA+VERA at ratio of 1:15 (drug/lipid) was calculated as 402.5 ± 36.53, 342.8 ± 47.50, and 502.4 ± 68.27 nm, respectively. This increase in the size of the drug-loaded NLCs was taken as an indication of successful encapsulation of the drugs, even at high drug/lipid ratios. Furthermore, for both ratios, the increase in the size of the dual drug-loaded NLCs indicates successful coencapsulation of the DOX-OA and VERA drugs. As a result of loading DOX-OA and VERA in all combinations, the PDI values remained below 0.24 and a low PDI suggests that the particles exhibit a high degree of monodispersity.^47^

**Table 1:** Variations in Size, EE%, and DL% of DOX-OA, VERA, and Dual Drug-Loaded (DOX-OA+VERA)@NLC Particles in Both 1:15 and 1:144 Ratios^a^

**(drug/lipid) formulation**	**particles**	**APIs**	**DLS (nm)**	**PDI**	**AFM (nm)**	**EE (%)**	**DL (%)**
1:15	**DOX-OA NLC**	**DOX-OA**	272.87 ± 38.15	0.19 ± 0.03	402.5 ± 36.53	93.50 ± 0.78	6.19 ± 0.27
	**VERA NLC**	**VERA**	289.36 ± 39.10	0.21 ± 0.05	342.8 ± 47.50	67.54 ± 1.22	5.90 ± 0.13
	**(DOX-OA+VERA)@NLC**	**DOX-OA**	362.22 ± 36.60	0.17 ± 0.03	502.4 ± 68.27	93.86 ± 0.54	6.21 ± 0.24
		**VERA**				64.19 ± 1.82	5.82 ± 0.21
1:144	**DOX-OA NLC**	**DOX-OA**	185.28 ± 27.57	0.24 ± 0.03	204.5 ± 32.75	95.32 ± 2.12	0.64 ± 0.04
	**VERA NLC**	**VERA**	166.82 ± 17.87	0.21 ± 0.02	170.0 ± 29.96	71.56 ± 0.02	0.94 ± 0.05
	**(DOX-OA+VERA)@NLC**	**DOX-OA**	203.04 ± 30.70	0.16 ± 0.03	320.8 ± 57.63	96.15 ± 2.16	0.98 ± 0.28
		**VERA**				73.42 ± 1.84	0.96 ± 0.32

a Data are presented as mean ± SD (*n* = 3).

When the EE and DL capacities were examined, it was found that DOX-OA, which has a more hydrophobic structure than VERA, can be better encapsulated. For dual drug-loaded NLCs, the encapsulation efficiencies of DOX-OA and VERA were calculated as 96.15 ± 2.16 and 73.42 ± 1.84% for the 1:114 (drug:lipid) ratio and 93.86 ± 0.54 and 64.19 ± 1.82% for the 1:15 (drug/lipid) ratio, respectively. The DL capacities determined the difference between these two drug loading ratios, which were very close to each other. For DOX-OA at a ratio of 1:144 (drug:lipid), the DL% was calculated as 0.98 ± 0.28% and at the ratio of 1:15 (drug:lipid), the DL% was calculated as 6.21 ± 0.24%. Similarly, for VERA, DL% was calculated as 0.96 ± 0.32 and 5.82 ± 0.21% at the ratios of 1:144 (drug:lipid) and 1:15 (drug:lipid) ratios, respectively. As the amount of drug-loaded per lipid was enhanced, the DL capacity of NLCs also increased significantly. Moreover, as expected, the more hydrophobic nature of DOX-OA explained its higher encapsulation in NLCs compared to VERA. As the amount of loaded drug increased, it was seen that NLCs would be able to encapsulate more drugs and thus reach the desired concentration more quickly when released from the NLCs. Due to these reasons and because the particle size was still suitable for intravascular administration, a ratio of 1:15 was more advantageous.

The EE of DOX NLC was reported as 82.75 ± 1.96% at a ratio of 1:15, our previous study.^40^ In this study, the encapsulation efficiency was increased by 11.5%, successfully using the ion-pairing mechanism. A comparable result was documented by Zhao et al., who tried to increase EE% with oleic acid ion-pairing DOX.^30^ NLC exhibits a high EE due to the strong affinity of the DOX-OA ion pair for the inner oil phase. NLCs composed of solid and liquid lipid disrupt the formation of lipid crystalline structures accompanied by an enhancement in defects, thereby causing minimization of the drug expulsion phenomenon and increased localization of more DOX-OA within the hydrophobic NLC core.^30^

To use AuNPs as agents for PTT, it is essential to consider their plasmonic properties, in particular, their absorption cross section and absorption efficiency. These properties determine the amount of thermal energy that can be conducted per particle.^48^ In this regard, AuNRs are good PTT agents that can be used in the 808 nm laser irradiation. Therefore, in this study, AuNRs were prepared by the seed-growth method and the sizes and geometries of AuNRs were examined by TEM. The longitudinal length of the AuNRs was determined to be 59.5 ± 28.94 nm, while the width was found to be 10.2 ± 3.34 nm (Figure S2). The aspect ratio was also determined to be 5.8. The aspect ratio is one of the most important parameters in terms of the photothermal conversion efficiency. In particular, it has been reported that AuNRs with long-thin geometry have better photothermal properties for the use of an 808 nm laser wavelength.^49^

The size variation of AuNRs after encapsulation in NLC was investigated by DLS and AFM. The size change was studied by gradually enhancing the concentration of AuNRs entrapped in the NLC and is given in Table S2. As the concentration of AuNRs increased from 19.5 to 52 μg Au/mg lipid, their sizes were calculated as 227.76 ± 81.78, 253.37 ± 43.35, and 302.41 ± 83.47 nm, respectively. A bright-field TEM image of AuNR-loaded NLCs at a concentration of 19.5 μg Au/mg lipid is shown in Figure S3. The NLCs, which initially exhibited a spherical morphology without gold loading, lost their fully spherical structure after incorporation of AuNRs. TEM analysis revealed that the AuNRs loaded with NLCs, which appeared more rectangular, measured 156.17 ± 58.95 nm in length and 114.54 ± 34.13 nm in width. In contrast, the size of the NLCs was found to be 43.68 ± 16.14 nm before AuNR encapsulation. The increase in size, the difference in geometry, and with the addition of gold, the absence of AuNRs on the outside prove that AuNRs were encapsulated in the NLC. In their study, Zheng et al. introduced polyhedral AuNPs into NLCs and conducted a size analysis.^50^ They reported that when AuNPs were incorporated into NLCs measuring 267.4 nm in size, the diameter increased to 365.5 nm. According to their findings, the size increment was attributed to the presence of solid AuNPs, which occupied significant space in the NLC matrix.

### Hyperthermia Study

**Figure 2:**
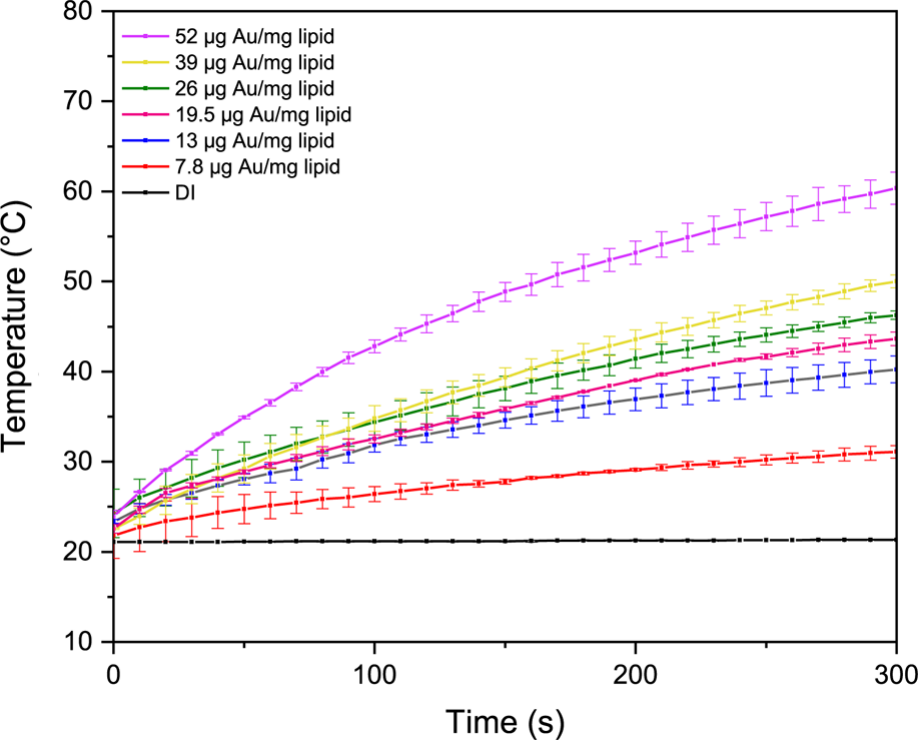
Temperature change profiles of NLC dispersions with varying concentrations of AuNRs (7.8–52 μg of Au/mg of lipid) under 808 nm NIR irradiation and 2.3 W power for 5 min. Data are presented as mean ± SD (*n* = 3).

Bare gold nanoparticles lack stability during the irradiation process, which prevents them from effectively harnessing enough energy to eliminate tumor cells.^51^ For this reason, a study was carried out to examine AuNRs by loading them into NLCs. Temperature change profiles for NLCs loaded with different concentrations of AuNRs were studied under 808 nm NIR irradiation at 2.3 W power for 5 min (Figure 2). The temperature of DI water, used as a control group, produced a temperature difference of 0.7 °C under these conditions. At a lower concentration (7.8 μg Au/mg lipid), the temperature reached 31.1 °C after 5 min. Thus, a temperature difference was calculated as 9.3 °C after 5 min. Hyperthermia causes irreversible cell damage by loosening cell membranes in the range of 41–47 °C.^52^ Therefore, the concentration was increased to achieve higher temperatures. Temperatures of 40.2, 43.6, 46.3, and 50.0 °C were recorded after 5 min of NIR irradiation at 13, 19.5, 26, and 39 μg Au/mg lipid concentrations, respectively. At a higher concentration (52 μg Au/mg lipid), the temperature reached 60.4 °C after 5 min of NIR irradiation and the temperature difference was calculated as 36.6 °C. The temperature of the AuNRs NLC increased dramatically with increasing concentration at constant irradiation time. After all, it is believed that the use of AuNRs at a concentration of 19.5 μg of Au/mg of lipid would be more advantageous as it is within a temperature range (43.6 °C) that does not induce apoptosis in healthy cells. Another critical parameter for ensuring safe cancer cell death is laser power. Figure S4 presents the temperature change profiles of AuNRs at a concentration of 19.5 μg of Au/mg of lipid exposed to varying laser power levels. The data show that as laser power decreases, the temperature difference also declines at the end of 5 min. In both clinical and preclinical applications, low-power laser treatments offer significant advantages by enhancing safety. However, it was observed that laser power levels below 2.3 W (∼1 W/cm^2^) were insufficient to induce the necessary temperature increase for effective cancer cell death. A power level of 2.3 W has been identified as the optimal power density, as it falls within the safe range established for *in vivo* and preclinical applications. Nevertheless, to further reduce laser power in future studies, adjustments to laser exposure time and photothermal agent concentration will be investigated.

### *In Vitro* Release Studies

The drug release experiment was conducted to assess the release profiles of DOX-OA and VERA from NLC. In the scope of work, DOX-OA and VERA release profiles from NLC were investigated in both 1:15 and 1:144 ratios, respectively. For this, DOX-OA and VERA were encapsulated separately and coloaded in the NLC. The pH differences were selected to simulate drug circulation in the bloodstream and also simulate the behavior within the cancerous cell environment. Also, the releases of DOX were examined to compare hydrophobicity difference between ion-pairing DOX and the free DOX from NLC (Figure S5). When analyzing DOX release from DOX NLC, the release reached 78.40 and 38.24% at the end of 48 h at pH 7.4, at ratios of 1:144 and 1:15 (drug/lipid), respectively. At the end of 1 h, DOX released 40.68% at the 1:144 (drug/lipid) ratio and 17.70% at the 1:15 (drug/lipid) ratio. Same experiments were repeated under the pH 5.5 condition. At this pH, the DOX release reached 88.62% at the end of 48 h, while after 1 h, the release was examined as 56.44% at 1:144 (drug/lipid) ratio and DOX release was calculated as 45.30% at the end of 48 h, while at the end of 1 h, the release reached 21.86% at the 1:15 (drug/lipid) ratio. An approximately 10% higher concentration was determined at pH 5.5. DOX exhibits varying solubilities depending on the pH level and its protonation state. As a result, DOX shows greater solubility and increased hydrophilicity at lower pH values. Consequently, DOX incorporated within the NLC tends to diffuse out of the nanoparticle matrix in a lower pH environment. An acidic cancerous cell environment may contribute to a higher concentration of DOX, significantly improving the efficacy of DDS.^53,54^

**Figure 3:**
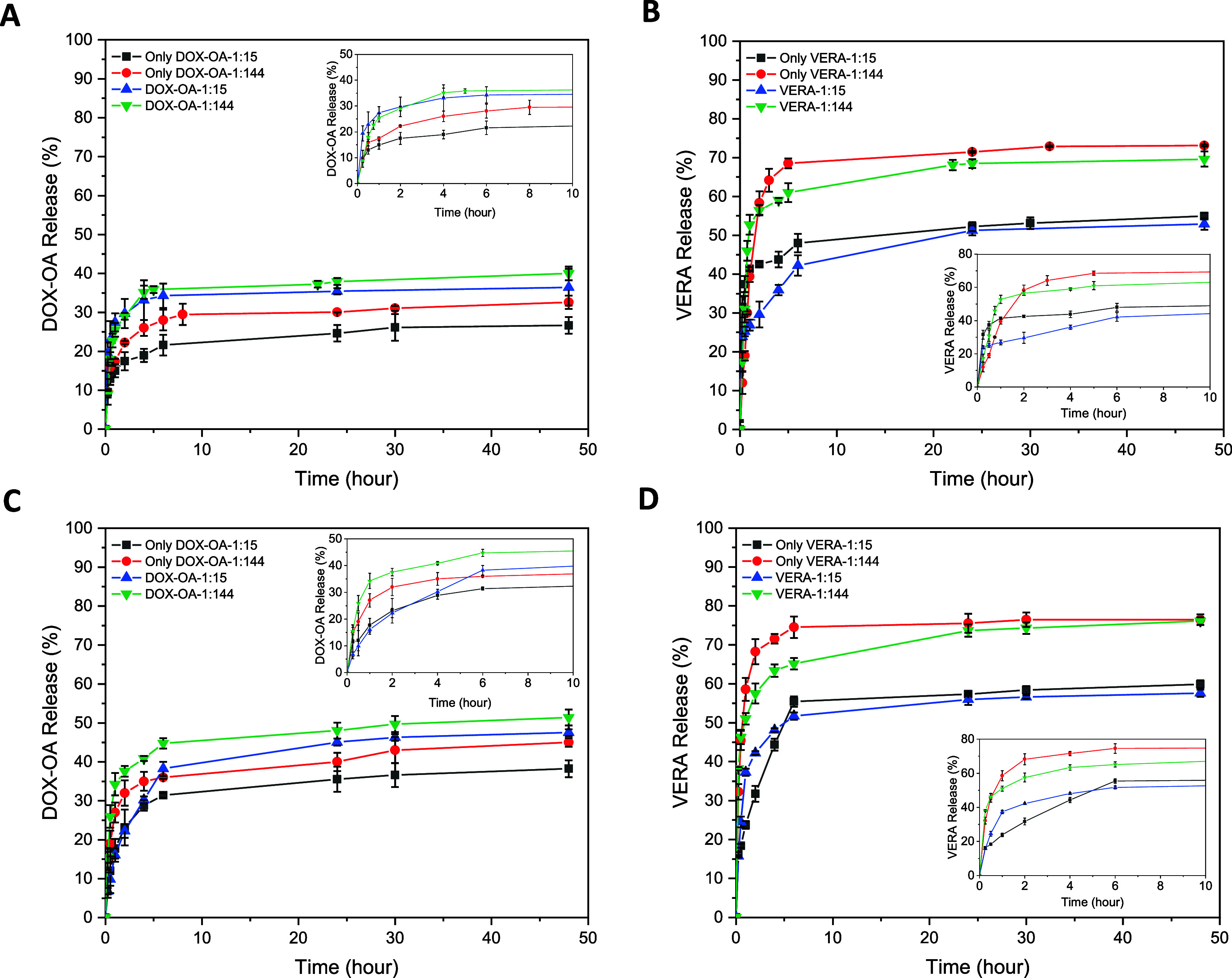
*In vitro* release profile of (A) DOX-OA from both DOX-OA NLC (only DOX-OA) and (DOX-OA+VERA)@NLC at pH 7.4, (B) VERA from both VERA NLC (Only VERA) and (DOX-OA+VERA)@NLC at pH 7.4, (C) DOX-OA from both DOX-OA NLC (Only DOX-OA) and (DOX-OA+VERA)@NLC at pH 5.5, and (D) VERA from both VERA NLC (Only VERA) and (DOX-OA+VERA)@NLC at pH 5.5 in the PBS solution (including 0.7% Tween-20 (w/v%)) over 48 h at 37 °C. Inset shows releases over a shorter time period (10 h). Data were presented as mean ± SD (*n* = 3).

Although a lower percentage release was observed at a 1:15 (drug/lipid) ratio for DOX-OA and VERA releases, concentration reached after 48 h has increased. At pH 7.4, DOX-OA release from DOX-OA NLC reached 26.64% at the end of 48 h (Figure 3A). Moreover, the DOX-OA concentration was calculated as 0.00106 mg/mL. After 1 h, release reached 14.97% and the concentration was calculated as 0.006 mg/mL. DOX-OA release from (DOX-OA+VERA)@NLC increased to 36.47% at the end of 48 h. The percentage of DOX-OA released reached 27.42% by the end of the first hour. An increased amount of DOX-OA was released into the medium from the coloaded NLCs. At pH 7.4, DOX-OA release from coloaded NLCs was 9.83% higher compared to DOX-OA alone. The release difference at a 1:144 (drug:lipid) ratio was examined as 7.37%. Notably, the difference increases as more APIs are incorporated into the NLCs, which is indicative of a greater encapsulation of drugs within the structure. In Figure 3C, we can see that the DOX-OA release from DOX-OA NLC was calculated as 38.24% at the end of 48 h, and the concentration was calculated as 0.00151 mg/mL at pH 5.5. Also, at the end of the first hour, the DOX-OA release was calculated as 17.70%. DOX-OA release from (DOX-OA+VERA)@NLC enhanced to 47.52% at the end of 48 h and was calculated as 16.04% after the first hour. A higher percent release of DOX-OA was noted from coloaded NLCs. After 48 h, DOX-OA release from coloaded NLCs was 9.28% higher than that of DOX-OA alone. The release from both DOX-OA NLCs and (DOX-OA+VERA)@NLC reached a higher concentration at pH 5.5. At low pH values, DOX-OA becomes unstable and undergoes protonation, leading to a faster release from the lipid matrix.

In addition, the releases of VERA from VERA NLC and (DOX-OA+VERA)@NLC were examined at pH 7.4 and pH 5.5 (Figure 3B,D). VERA release from VERA NLC was calculated as 59.85% at the end of 48 h, while the release reached 23.80% at the end of 1 h at pH 5.5. VERA release from NLCs coloaded with DOX-OA and VERA reached 57.59% after 48 h, while the release was 37.30% after the first hour. The release of DOX-OA from the coloaded NLCs showed a significant change, whereas the percentage release of VERA remained relatively unaffected. In addition, the VERA release slightly increased at low pH values. Comparing the pH impact on DOX-OA and VERA release, DOX-OA was more influenced by the lower pH compared to VERA in the ratio of 1:15 (drug/lipid). As previously mentioned, this is linked to the increased solubility of DOX-OA due to protonation at low pH. However, for VERA, the solubility difference at low pH does not significantly impact its release across all ratios. VERA exhibited higher release concentrations compared to those of DOX-OA at all time intervals. As previously noted, VERA is an API used to resensitize cancer cells that have developed resistance.^16^ If the concentration of VERA released exceeds that of DOX-OA, then the effectiveness of chemotherapy is expected to improve. The coloaded NLC at a ratio of 1:15 (drug/lipid) is advantageous in terms of high DL capacity and reduced burst release. For this reason, in order to obtain a sufficient dose for chemotherapy and sustained release, this study continued with coloaded NLCs at a 1:15 (drug/lipid) ratio.

**Figure 4:**
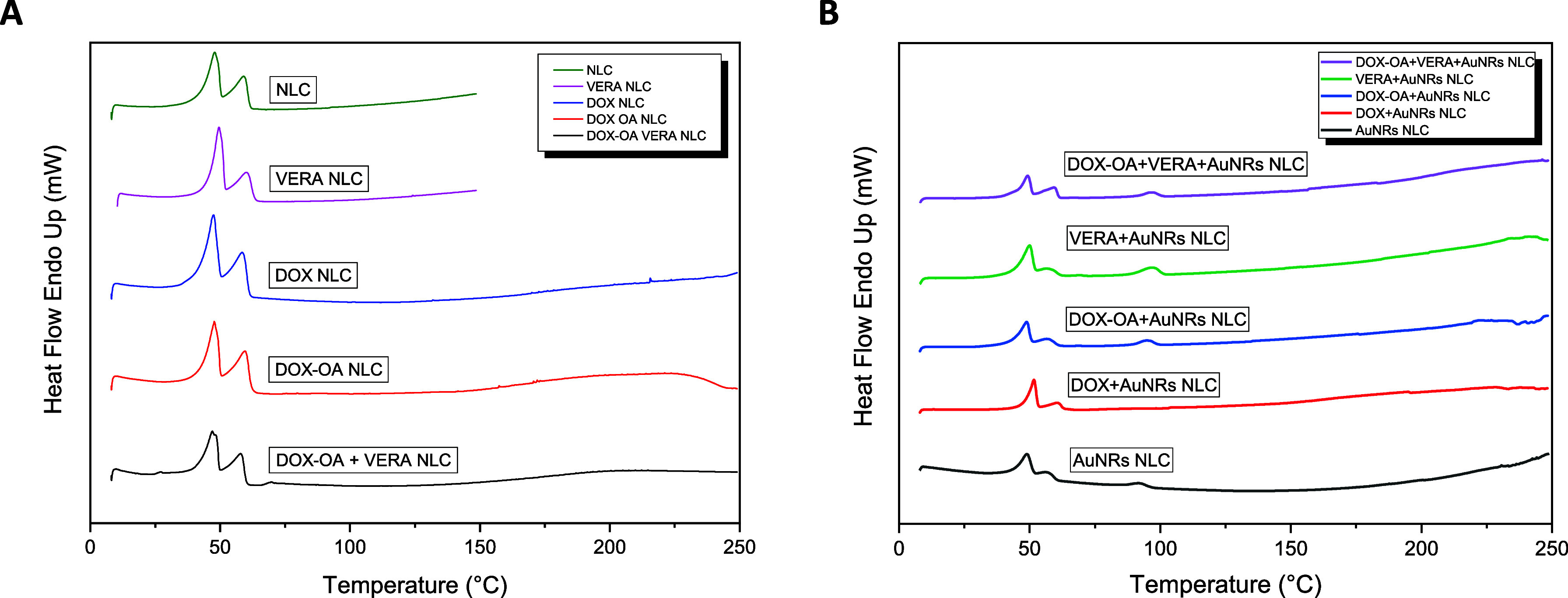
Different combinations include DSC thermograms of (A) NLC, VERA NLC, DOX NLC, DOX-OA NLC and DOX-OA+VERA NLC and (B) AuNRs NLC, DOX+AuNRs NLC, DOX-OA+AuNRs NLC, VERA+AuNRs NLC, and DOX-OA+VERA+AuNRs NLC. Samples were prepared with weights ranging from 12.6 to 21.9 mg. Measurements were carried out 10–250 °C at a 10 °C/min speed.

DSC analysis was used to investigate the crystal structures of coloaded NLCs and also to understand whether the APIs or AuNRs were encapsulated in the NLCs. The enhancement in the temperature was in the range of 10 to 250 °C, at a rate of 10 °C/min, applied for different combinations of NLCs. In this study, a wider temperature range was selected, because free APIs melt at higher temperatures. To analyze the difference between drug-loaded NLCs and free drugs in the medium, and to check whether encapsulation is taking place efficiently, this method was preferred. In Figure 4A, we can clearly see two different peaks. The melting of the surfactant shell and the melting of lipid core create these peaks.^20^ The melting temperatures of DOX and VERA are known around 240 and 140 °C, respectively.^55,56^ The absence of these peaks at higher temperatures proves that drugs may be trapped in the NLCs.

The crystallinity index of NLC was determined to be 13.9% (Table S3). Encapsulation of DOX slightly increased the crystallinity index to 14.3%. In contrast, the addition of DOX-OA to the NLC reduced the crystallinity index to 11.5%. The crystallinity difference between DOX and DOX-OA stems from two key reasons. One reason is that the free DOX consists of both crystalline and amorphous structures. Thus, when encapsulated into the relatively amorphous NLC, there was minimal crystallinity difference observed due to its combined amorphous and crystalline properties. Conversely, due to its hydrophobic nature, DOX-OA was more efficiently loaded into the lipophilic NLC and contributed to reducing the crystallinity index further. When comparing DSC thermograms of the pure NLCs with DOX-OA NLC, it was observed that the endothermic peak of DOX-OA NLC appears more distinct. This observation implies the addition of DOX-OA created more defined liquid oil compartments within the solid matrix of NLCs. Such a shift could enhance the solubility of the drug and consequently boost the overall DL capacity.^30^ Also, previous studies have demonstrated that employing the ion-pairing technique can effectively preserve the drug encapsulated within the nanocarrier following intravenous administration, leading to enhanced pharmacokinetic properties.^57−59^ Besides, the crystallinity index of VERA was reduced more than those of both DOX and DOX-OA. CI% for VERA was calculated as 9.8% and was decreased slightly to 9.7% with the coloading into the NLCs. The lowest crystallinity was seen with coloading. This is one of the proofs that DOX and VERA were entrapped in the NLCs successfully.

Three characteristic peaks are seen in Figure 4B. Peaks indicate surfactant shell melting, stearic acid melting, and melting of excess CTAB that was not separated totally from the washing process, respectively. In addition, the absence of characteristic peaks associated with DOX, DOX-OA, VERA, and AuNRs indicates they were encapsulated within the NLCs.

The crystallinity index of AuNR NLC, DOX+AuNRs NLC, DOX-OA+AuNRs NLC, VERA+AuNRs NLC, and DOX-OA+VERA+AuNR NLC was calculated as 4.22, 1.36, 2.90, 3.13, and 3.05%, respectively (Table S4). Crystallinity index values decreased significantly as gold nanoparticles were added to the NLC structure. At the same time, it has been seen in previous studies that the EE and DL capacity of NLC increase when gold nanoparticles are added to the NLC. This information supports the results obtained from DSC. When crystalline gold nanoparticles were loaded into the amorphous NLC structure, a synergistic effect with the drugs was observed, resulting in a shift toward a more amorphous character.

Furthermore, the lowest crystallinity index among the coloaded NLCs was observed in AuNRs. Increased dissolution is achievable since materials in amorphous form exhibit higher saturation solubility compared to their crystalline counterparts. It is also well known that particles with amorphous characteristics enhance EE and improve drug retention stability. AuNRs have demonstrated compatibility with drug-loaded NLCs, in addition to their advantages as PTT agents. Based on the DSC studies, it is suggested that AuNRs may enhance long-term drug retention stability by imparting amorphous characteristics at low concentrations.

**Figure 5:**
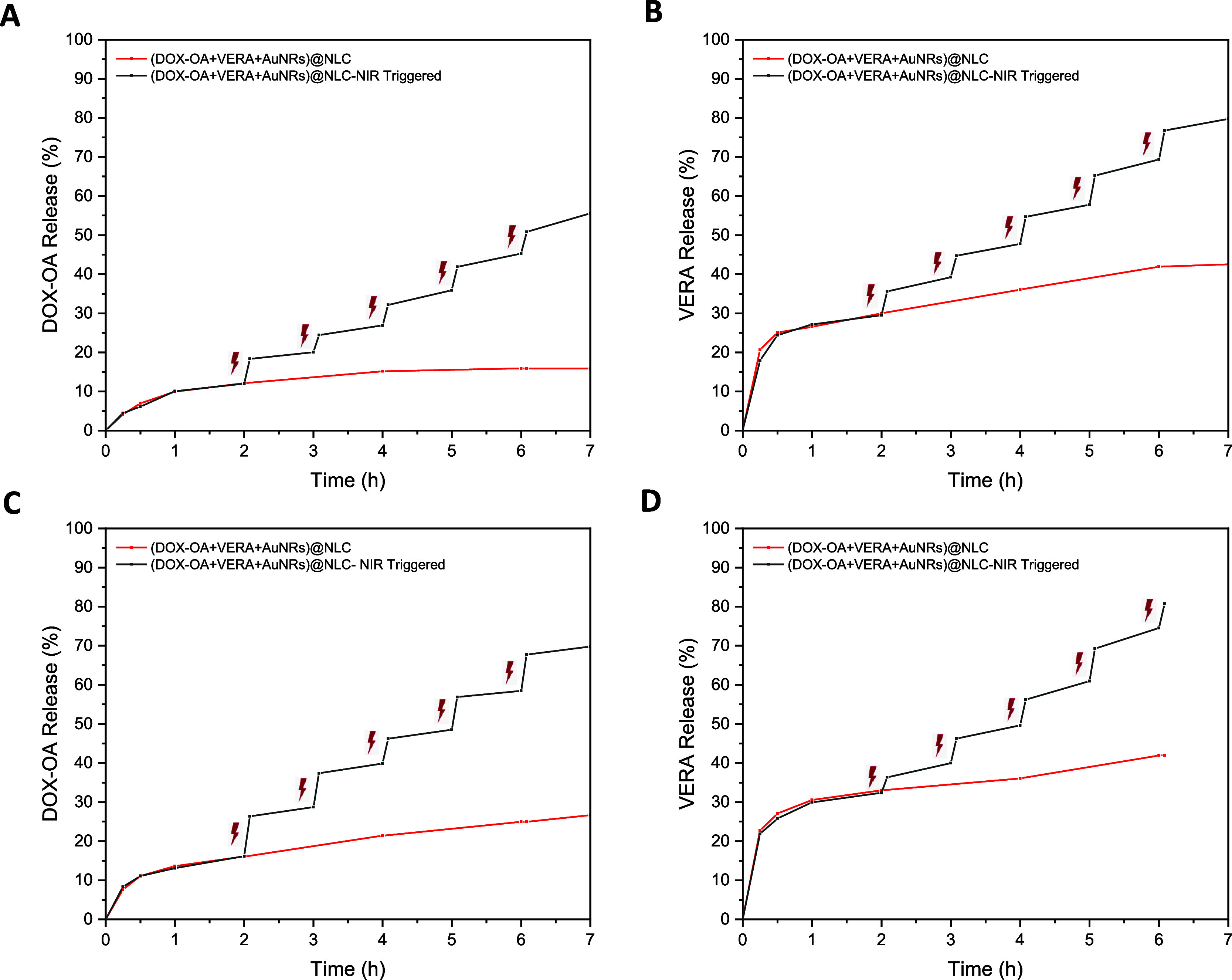
*In vitro* release profile of (A) DOX-OA and (B) VERA from and (DOX-OA+VERA+AuNRs)@NLC with and without NIR laser irradiation in PBS solution at 7.4 pH and (C) DOX-OA and (D) VERA from and (DOX-OA+VERA+AuNRs)@NLC with and without NIR laser irradiation in PBS solution at 5.5 pH (including 0.7% Tween-20 (w/v%)) at 37 °C. Data were presented as mean ± SD (*n* = 3).

DOX-OA and VERA releases from (DOX-OA+VERA+AuNRs)@NLC were examined with and without NIR laser irradiation. DOX-OA and VERA percent releases were enhanced with NIR laser irradiation regardless of the pH value. DOX-OA release and VERA were calculated as 50.8 and 76.7%, respectively, at the end of the fifth cycle at pH 7.4 (Figure 5A,B). While VERA was more hydrophilic than DOX-OA, the percent release of VERA was higher than that of DOX-OA. The temperature of (DOX-OA+VERA+AuNRs)@NLC was detected as 52.1 °C at the end of the fifth cycle. DOX-OA and VERA releases were calculated as 67.7 and 80.8%, respectively, at the end of the last cycle at pH 5.5 (Figure 5C,D). Cationic DOX was released at higher pH values. Because of that reason, DOX can reach a higher concentration at 5.5, the pH value of the tumor region. The unique “stepped” pattern observed in the drug release profile indicates that the release of the drug can only be initiated by NIR exposure. Moreover, the quantity of released drug is strongly influenced by both the duration and intensity of the NIR exposure. This demonstrates the achievement of “NIR-light-controlled precise drug release”, where the release of the drug can be finely regulated by 808 nm NIR light.

After laser irradiation, the changes in the structure of NLCs were wondered. Therefore, the NLC DDS was analyzed using bright-field TEM imaging to investigate how it is affected by the temperature following NIR laser irradiation. For this purpose, the morphology and size changes of NLCs with and without NIR irradiation were examined. AuNRs NLC has a rectangular-like morphology that is evident on a large surface (Figure S6.A). In previous studies, the size of AuNRs NLC was analyzed to be 156.17 ± 58.95 nm in length and 114.54 ± 34.13 nm in width. The melting temperature of AuNRs NLC was also calculated as 59.42 °C according to the DSC analysis. Also, according to the TEM image of the particles at the end of the fifth cycle of NIR laser irradiation, the (DOX-OA+VERA+AuNRs)@NLC in the medium has decreased significantly (Figure S6.B). As a result of all these characteristic features, the AuNPs in the rod geometry provided an advantage in the degradation of NLC.

Mathematical modeling was performed in order to better analyze the release behavior of the APIs. Kinetic models such as “zero order model”, “first order model”, “Higuchi model”, “Korsmeyer–Peppas model”, and “Hixson-Crowell model” were used in the scope of this study. The parameters (*R*^2^, *k*, *n*) are introduced in Table 2. Kinetic release plots for each APIs are also given in Figures S7 and S8 for DOX-OA and VERA release from nonencapsulated AuNRs NLC at pH 5.5 and pH 7.4, respectively, and given in Figure S9 for dual drug and AuNR-loaded NLC at both pH values. When five different models were examined for DOX-OA release from DOX-OA NLC, VERA release from VERA NLC, and DOX-OA and VERA release from coloaded NLC, the “Korsmeyer-Peppas kinetic model” fits with 0.9399, 0.8725, 0.9009, and 0.8633 regression values, respectively, at pH 7.4. The “Korsmeyer-Peppas model” relates drug release exponentially to elapsed time, using *n* values to characterize various release mechanisms. The *n* value, diffusion exponent, may be used for characterizing the mechanism of different release kinetics. In this study, *n* values are given as 0.6206, 0.8248, 0.7404, and 0.7369 for DOX-OA NLC, VERA NLC, DOX-OA, and VERA release from (DOX-OA+VERA)@NLC, respectively. For the range “0.45 < *n* < 0.89”, the release fits to non-Fickian diffusion. According to this information, all APIs were at a range of non-Fickian transport. Diffusion and erosion or relaxation may occur simultaneously for this type of transport. On the other hand, DOX-OA and VERA releases from DOX-OA NLC and VERA NLC fit the “Higuchi model” with regressions of 0.9744 and 0.9692, respectively. Higuchi model describes drug dissolution from various modified-release pharmaceuticals. In addition, it is known that the Higuchi model defines the properties of drug release on Fick’s Law as a diffusion process based. This suggests that the release mechanism is primarily diffusion-controlled, consistent with the characteristics of matrix-based DDSs. The linear relationship between drug release and the square root of time further supports the sustained release behavior observed in this study. Besides, the release of DOX-OA and VERA from dual drug-loaded NLC follows the Korsmeyer-Peppas model with *R*^2^ values of 0.9966 and 0.9634, respectively. *n* values of DOX-OA and VERA were suitable for non-Fickian diffusion. The *n* values were calculated as 0.6568 for DOX-OA and calculated as 0.8178 for VERA, that is, the release kinetics from coloaded NLCs, regardless of the ambient pH for this study. Also, DOX-OA and VERA release kinetic modeling was performed from rod-shaped gold nanoparticles and dual drug-loaded NLCs. The release of DOX-OA and VERA appears to always fit the Korsmeyer-Peppas model at pH 7.4 and pH 5.5. *R*^2^ values for DOX-OA and VERA release were calculated as 0.9929 and 0.8868 for pH 5.5 and 0.9621 and 0.8886 for pH 7.4, respectively. The *n* values are also given as 0.5391 and 0.7404 at pH 5.5 and 0.6022 and 0.7645 at pH 7.4 for the DOX-OA and VERA, respectively. The release is consistent with non-Fickian diffusion when the range is 0.45 < *n* < 0.89. Regardless of the changing pH and the presence of AuNRs in the structure, the release kinetics of the coloaded NLC always followed the Korsmeyer-Peppas model. Additionally, the presence of AuNRs in the structure enhances the solubility of DOX and VERA by increasing the temperature under NIR laser irradiation. This temperature increase also contributes to an increase in the diffusion coefficient. The increasing diffusion coefficient after each cycle, peaking at the fifth cycle, indicates that DOX and VERA release under NIR laser irradiation are also diffusion-dependent. The (DOX-OA+VERA+AuNRs)@NLC, which follows the Korsmeyer-Peppas model in the absence of NIR laser irradiation, also exhibits a diffusion-dependent mechanism under NIR laser irradiation, with concentration increasing at a faster rate over time.

**Table 2:** Release Kinetic Parameters of DOX-OA NLC, VERA NLC, (DOX-OA+VERA)@NLC, and (DOX-OA+VERA+AuNRs)@NLC at pH 5.5 and 7.4

		**DOX-OA NLC**		**VERA NLC**		**(DOX-OA+VERA)@NLC**				**(DOX-OA+VERA+AuNRS)@NLC**			
						**pH 5.5**		**pH 7.4**		**pH 5.5**		**pH 7.4**	
**kinetic model**	**parameters**	**pH 5.5**	**pH 7.4**	**pH 5.5**	**pH 7.4**	**DOX-OA**	**VERA**	**DOX-OA**	**VERA**	**DOX-OA**	**VERA**	**DOX-OA**	**VERA**
**zero order**	**R**^**2**^	0.8151	0.6700	0.7881	0.4749	0.9226	0.8022	0.5852	0.4519	0.8345	0.5335	0.7317	0.5395
	**k**	0.1640	0.1173	0.2193	0.2561	0.1728	0.3207	0.1904	0.1700	0.0920	0.1834	0.1128	0.2051
**first order**	**R**^**2**^	0.8479	0.6944	0.8391	0.5244	0.9420	0.8511	0.6275	0.4826	0.8474	0.5730	0.7535	0.5838
	**k**	–0.0008	–0.0012	–0.0012	–0.0015	–0.0009	–0.0019	–0.0010	–0.0009	–0.0004	–0.0010	–0.0005	–0.0011
**Higuchi**	**R**^**2**^	0.9744	0.9692	0.9692	0.7799	0.9935	0.9672	0.8643	0.7499	0.9808	0.8226	0.9511	0.8306
	**k**	2.0687	2.8057	2.8057	3.7853	2.0686	4.0618	2.6698	2.5265	1.1510	2.6276	1.4831	2.9357
**Korsmeyer-Peppas**	**R**^**2**^	0.9533	0.9375	0.9375	0.8725	0.9966	0.9634	0.9009	0.8633	0.9929	0.8868	0.9621	0.8886
	**k**	0.850	0.1134	0.1134	0.1895	0.0205	0.0917	0.1476	0.1765	0.0065	0.1590	0.0690	0.1628
	**n**	0.6646	0.7350	0.7350	0.8248	0.6568	0.8178	0.7404	0.7369	0.5391	0.7404	0.6022	0.7645
**Hixson-Crowell**	**R**^**2**^	0.7563	0.7623	0.7623	0.4877	0.8653	0.8052	0.5704	0.4458	0.7169	0.5227	0.6496	0.5349
	**k**	0.0031	0.0042	0.0042	0.0051	0.0032	0.0062	0.0036	0.0033	0.0018	0.0035	0.0022	0.003

Indeed, it was expected that the Korsmeyer-Peppas model would provide the most accurate fit for the anomalous drug release data collected in this study. The early phase of drug release results from diffusion from lipid-based nanoparticles. However, the drug remaining within the nanocompartments was released only as the lipid undergoes degradation. The main aim of the study is to use NIR laser irradiation to raise the drug concentration in the tumor microenvironment. This allows for sustained release from lipid particles that will degrade upon an increase in temperature. Similarly, in their study, Jusu et al. examined the controlled release and kinetic model of polymer-based PLGA-CS-PEG microparticles for the treatment of TNBC cells.^60^ They stated that the release kinetics of these temperature-sensitive polymer-based microparticles follows anomalous non-Fickian diffusion; thus, the drug was released by degradation.

### *In Vitro* Studies with MDR-Resistant MDA-MB-231 Cells

**Figure 6:**
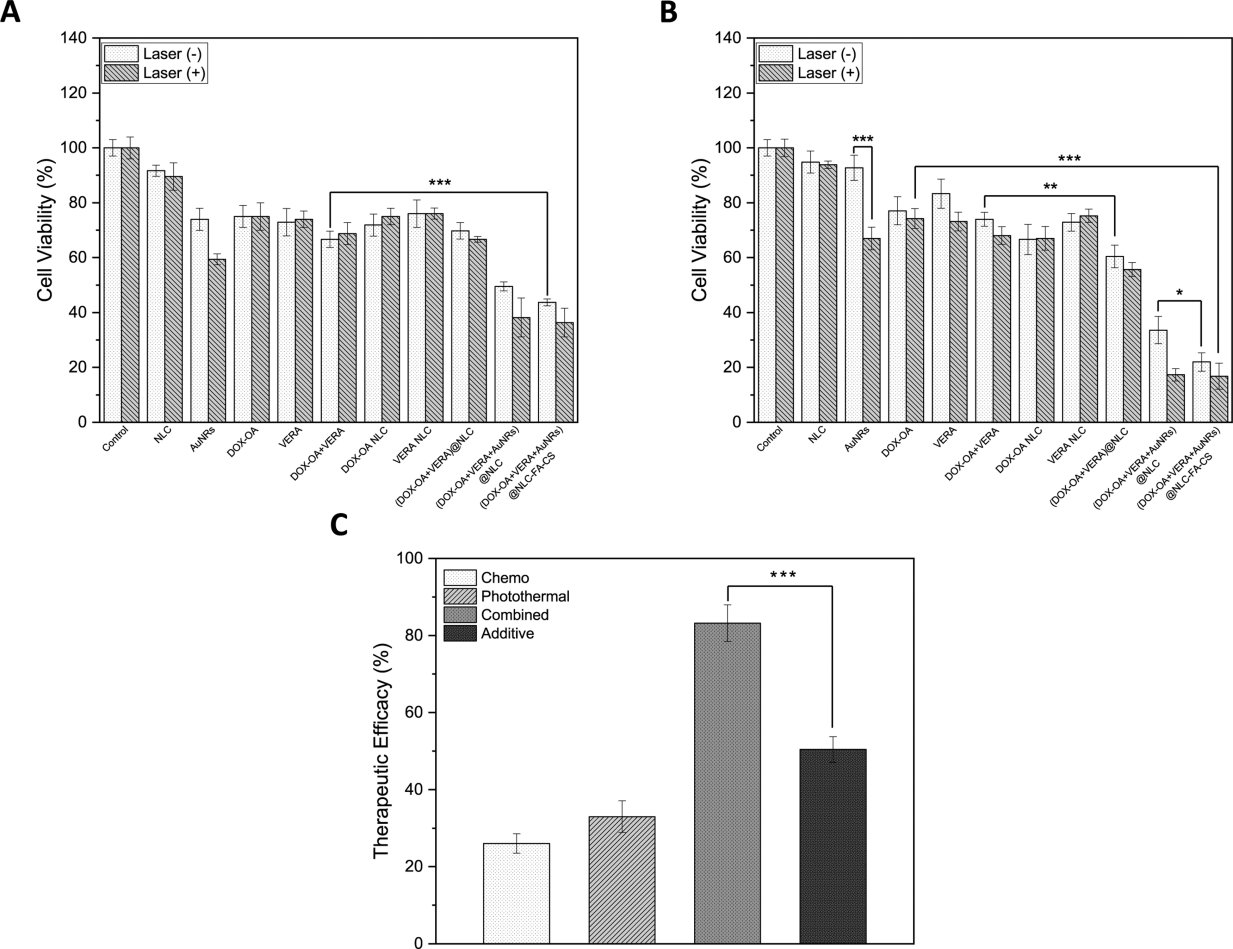
Percent cell viability of the control group and the experimental groups with/without 808 nm NIR laser application at 2.3 W power on MDA-MB-231^R^ cells (A) at 24 h and (B) at 48 h. (C) Therapeutic efficacies of MDA-MB-231^R^ cells treated with DOX-OA+VERA (Laser(−)), AuNRs (Laser(+)), and (DOX-OA+VERA+AuNRs)@NLC-FA-CS with NIR laser and additive therapeutic efficacy. Data were presented as mean ± SD. Statistical analysis was conducted using one-way ANOVA with Tukey’s multiple-comparisons post-test comparing all conditions. Statistical significance is indicated with *p < 0.05, **p < 0.01, ***p < 0.001.

Cell viability assays were performed on MDA-MB-231^R^ cells to examine the cytotoxic effects of free APIs, AuNRs, and the NLC formulations in the presence and absence of 808 nm NIR irradiation (Figure 6A,B). First, it is seen that AuNRs did not show a significant toxicity on the MDA-MB-231^R^ cell line without NIR irradiation. However, when laser is applied, 67.01% increase in cell killing ability can be achieved, which would be a powerful tool to increase the ultimate therapeutic efficacy. When DOX-OA and DOX-OA+VERA results were compared, regardless of the laser irradiation, it is clear that using VERA as a chemosensitizer is not enough if the administration of the drug mixture was kept conventionally. From the 48 h results (Figure 6B), it is observed that application of neither DOX-OA, VERA nor DOX-OA+VERA created a sufficient therapeutic efficacy to combat MDR-resistant cancer cell proliferation. Despite this, it is seen that entrapment of the DOX-OA+VERA combination into NLCs allowed observation of a statistically significant difference in terms of cell viability (Figure 6B). As it is mentioned before, encapsulation of DOX-OA and VERA into NLCs provides the regulation of the drug release rates and order, which is the key point in terms of first resensitizing the MDR-resistant cancer cells and then inhibiting their growth. In addition to that, as is clearly seen in Figure 6B, encapsulation of AuNRs besides DOX-OA+VERA ((DOX-OA+VERA+AuNR)@NLC) and application of NIR irradiation created a remarkable difference in killing the cancer cells when compared with (DOX-OA+VERA)@NLC by decreasing the cell viability to 17.30%. (For IC50 values of the formulations, see Supporting Information Table S5). When it comes to revealing the effect of FA-CS surface coverage on the cell internalization of NLC formulations, (DOX-OA+VERA+AuNR)@NLC and (DOX-OA+VERA+AuNR)@NLC-FA-CS were compared with each other. It is observed that without NIR laser irradiation, FA-CS-covered NLC formulations can exhibit 11.6% more cell killing ability than uncovered ones can (48 h). Moreover, in just 24 h, the effect of using NLC as the drug carrier and FA-CS for surface coverage can be counted as significant when compared with the physical mixture of DOX-OA and VERA, which emphasizes that the strategy used in this study is conceptually successful (Figure 6A). Since the cancer cell can sustain a low pH environment, which promotes the protonation of FA-CS, the specific distribution of particles could inhibit the growth of MDA-MB-231^R^ cancer cells that overexpress the folate receptor. It is widely reported that nanomaterials can be internalized into the cell and increase therapeutic potential.^61^ Yet, indiscriminate uptake by cells can result in toxicity to healthy cells and diminish the effectiveness of cancer treatment. Based on these results, coating NLCs with FA-CS was predicted to enhance specific targeting of cells by targeting FA receptors and facilitating enhanced cellular uptake.

As shown in Figure 6C, various therapeutic efficacies were compared to evaluate whether the system has the synergistic effect of chemotherapy and PTT. One of these various values, additive therapeutic efficacy (*T*_additive_), was calculated according to the equation: *T*_additive_ = 100 – (*f*_chemo_ × *f*_photothermal_) × 100 where *f* is the surviving cell fraction after each treatment.^62,63^*T*_additive_ was calculated as 50.4 ± 3.3%. In comparison to sole chemotherapy (DOX-OA+VERA (Laser(−)), 26.0%) and sole PTT (AuNRs (Laser(+)), 33.0%), *T*_additive_ was higher. On the other hand, *T*_additive_ was significantly lower than the calculated therapeutic efficacy of the group of (DOX-OA+VERA+AuNRs)@NLC-FA-CS with NIR laser (83.2 ± 4.6%) (*T*_combined_). These *in vitro* studies show the importance of the synergistic effect of chemo/PTT.

**Figure 7:**
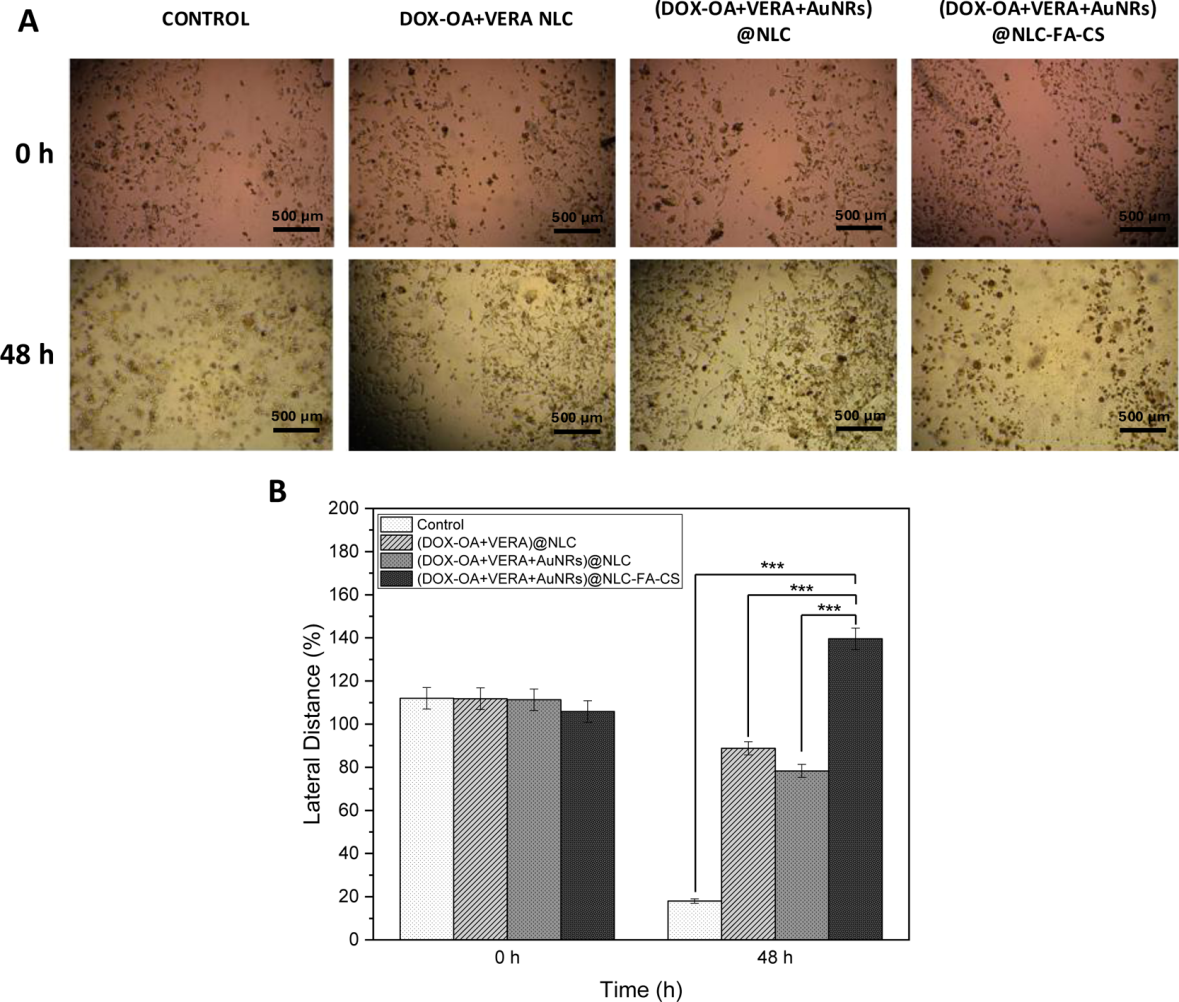
(A) Wound healing images after scratch incubation with DDSs free, DOX-OA+VERA NLC, (DOX-OA+VERA+AuNRs)@NLC, and (DOX-OA+VERA+AuNRs)@NLC-FA-CS for 48 h and (B) effects of DOX-OA+VERA NLC, (DOX-OA+VERA+AuNRs)@NLC and (DOX-OA+VERA+AuNRs)@NLC-FA-CS on lateral distance of MDA-MB-231 cells. Data were presented as mean ± SD. Statistical analysis was conducted using one-way ANOVA with Tukey’s multiple-comparisons post-test comparing all conditions. Statistical significance is indicated with *p < 0.05, **p < 0.01, ***p < 0.001.

Understanding the effects of NLCs on cancer cell collective migration, a scratch assay was conducted. After a “scratch” or “wound” was created in a cell culture, the cancer cells moved collectively into the empty area. Wound healing images were taken immediately and 48 h after the scratch. The microscope images of three different groups (DOX-OA+VERA NLC, (DOX-OA+VERA+AuNRs)@NLC, and (DOX-OA+VERA+AuNRs)@NLC-FA-CS are given in Figure 7A, and the average of lateral distance measured from wound healing images is shown in Figure 7B. Statistically, (DOX-OA+VERA+AuNRs)@NLC-FA-CS has significantly different wound-healing abilities compared with DOX-OA+VERA NLC and (DOX-OA+VERA+AuNRs)@NLC.

CS is the deacetylated derivative of chitin that is used extensively in drug technology.^64^ For lipid-based nanoparticles, the ammonium group of CS interacts with negatively charged groups in the construct. The ammonium group in CS may also be an attractant to lipids.^65^ The chitin-coated formulations exhibit good biocompatibility *in vivo*, which may be due to high uptake of lipid-based nanoparticles. It is shown that the chitin-coated formulations significantly improve the apoptosis rate in breast cancer cells compared to other formulations.^66^ In a study where 5-fluorouracil (5-FU)-loaded liposomes coated with chitin were developed to target colon cancer cells, it is shown that chitin enhanced the stability and sustained the release of 5-FU and increased the cytotoxicity of 5-FU.^67^ In addition, in the formulation used, CS was functionalized with FA.

FA is excessively used by cancer cells due to their active metabolism and high proliferation capacity.^68^ FA receptors (FRα, FRβ, and FRγ) are cysteine-rich glycoproteins present on the cell surface, which bind folate with a high affinity to facilitate its cellular uptake. In most cancer cells, FA receptors, especially FRα, are expressed at a very high level in order to meet the FA needs of these rapidly proliferating cells even at low FA availability. Therefore, it is frequently used as a targeting molecule for a variety of cancer cells. Its wide therapeutical and diagnostic use includes administration of anti-FRα antibodies, folate-based imaging agents, high-affinity antifolates, and folate-conjugated drugs and toxins.^69,70^ Here, it is aimed to deliver the drugs to cells via FA receptors that will bind and effectively take up the agent via its FA content. Here, we have found that the combined use of DOX-OA and VERA reduced the metastatic properties of cancer cells and caused effective cell death in the resistant MDA-MB-231 cell line. In addition, modification of the surfaces of NLCs with FA-CS also significantly increased the level of cell death. Once again, it is observed that the FA-CS coating enhances the cellular uptake potential of NLCs, allowing them to be targeted to tumor cells and maximizing the therapeutic efficacy of the carrier.

## Conclusions

To conclude, this study offers a dual chemo/PTT approach using all-in-one (DOX-OA+VERA+AuNRs)@NLCs. The loading efficiency of the NLC structure was increased by making the ion-pair mechanism of DOX, which is used as a chemotherapeutic agent. Also, co-encapsulating chemosensitizer drug VERA with DOX-OA was an effective choice for overcoming MDR. The presence of AuNRs in the NLC allowed hyperthermia and increased the temperature of the particles to 43.4 °C at 19.5 μg Au/mg lipid concentration, which is the appropriate temperature range for PTT. Also, the percent release of DOX-OA and VERA was increased at each step with (DOX-OA+VERA+AuNRs)@NLC triggered with NIR laser irradiation. The DSC thermograms and bright-field TEM images demonstrate that NIR laser application causes NLC degradation, leading to API release. The cell viability of the MDA-MB-231^R^ decreased below 20% with NIR irradiation. Considering its multifunctional, smart, and state-of-the-art features, this DDS shows promising potential as an alternative to traditional DDSs. Its dual-drug delivery facilities can overcome MDR, and its hyperthermia ability can be utilized for PTT. In future studies, the inclusion of *in vivo* experiments will be essential to validate the effectiveness and safety of the developed nanocarrier system in more complex biological environments. While *in vitro* models provide valuable insights, *in vivo* studies will offer a more comprehensive understanding of the system’s pharmacokinetics, biodistribution, and therapeutic potential. This step is crucial for translating the findings into clinical applications, ultimately improving treatment strategies for aggressive cancers like TNBC.

## Supporting Information Available

Size analysis of all particles (Table S1 and Figure S1), TEM image of AuNRs (Figure S2), size change values of AuNRs@NLC in different concentrations (Table S2), TEM image of AuNRs@NLC (Figure S3), temperature profile (Figure S4), release profile of DOX (Figure S5), DSC analysis (Table S3 and S4), TEM image of AuNRs@NLC with and without NIR irradiation (Figure S6), release kinetic model profiles (Figure S7, S8, S9), IC50 of the formulation (Table S5) (PDF)

## References

[ref1] Xia C.; Dong X.; Li H.; Cao M.; Sun D.; He S.; Yang F.; Yan X.; Zhang S.; Li N.; Chen W. Cancer statistics in China and United States, 2022: profiles, trends, and determinants. Chin. Med. J. 2022, 135, 584–590. 10.1097/CM9.0000000000002108. 35143424 PMC8920425

[ref2] Torre L. A.; Bray F.; Siegel R. L.; Ferlay J.; Lortet-Tieulent J.; Jemal A. Global cancer statistics, 2012. CA: a cancer journal for clinicians 2015, 65, 87–108. 10.3322/caac.21262. 25651787

[ref3] Lovitt C. J.; Shelper T. B.; Avery V. M. Doxorubicin resistance in breast cancer cells is mediated by extracellular matrix proteins. BMC Cancer 2018, 18, 1–11. 10.1186/s12885-017-3953-6. 29304770 PMC5756400

[ref4] Sparano J. A.; Gray R. J.; Makower D. F.; Pritchard K. I.; Albain K. S.; Hayes D. F.; Geyer C. E. Jr.; Dees E. C.; Goetz M. P.; Olson J. A. Jr.; Lively T.; Badve S. S.; Saphner T. J.; Wagner L. I.; Whelan T. J.; Ellis M. J.; Paik S.; Wood W. C.; Ravdin P. M.; Keane M. M.; Gomez Moreno H. L.; Reddy P. S.; Goggins T. F.; Mayer I. A.; Brufsky A. M.; Toppmeyer D. L.; Kaklamani V. G.; Berenberg J. L.; Abrams J.; Sledge G. W. Adjuvant chemotherapy guided by a 21-gene expression assay in breast cancer. N. Engl. J. Med. 2018, 379, 111–121. 10.1056/NEJMoa1804710. 29860917 PMC6172658

[ref5] Li S.; Zhao Q.; Wang B.; Yuan S.; Wang X.; Li K. Quercetin reversed MDR in breast cancer cells through down-regulating P-gp expression and eliminating cancer stem cells mediated by YB-1 nuclear translocation. Phytotherapy research 2018, 32, 1530–1536. 10.1002/ptr.6081. 29635751

[ref6] Ma W.; Chen Q.; Xu W.; Yu M.; Yang Y.; Zou B.; Zhang Y. S.; Ding J.; Yu Z. Self-targeting visualizable hyaluronate nanogel for synchronized intracellular release of doxorubicin and cisplatin in combating multidrug-resistant breast cancer. Nano Research 2021, 14, 846–857. 10.1007/s12274-020-3124-y.

[ref7] Zhang H.; Jiang W.; Liu R.; Zhang J.; Zhang D.; Li Z.; Luan Y. Rational design of metal organic framework nanocarrier-based codelivery system of doxorubicin hydrochloride/verapamil hydrochloride for overcoming multidrug resistance with efficient targeted cancer therapy. ACS Appl. Mater. Interfaces 2017, 9, 19687–19697. 10.1021/acsami.7b05142. 28530401

[ref8] Szakács G.; Paterson J. K.; Ludwig J. A.; Booth-Genthe C.; Gottesman M. M. Targeting multidrug resistance in cancer. Nat. Rev. Drug Discovery 2006, 5, 219–234. 10.1038/nrd1984. 16518375

[ref9] Gillet J.-P.; Gottesman M. M. Mechanisms of multidrug resistance in cancer. Multi-drug resistance in cancer 2010, 596, 47–76. 10.1007/978-1-60761-416-6_4. 19949920

[ref10] Li X.; Jia X.; Niu H. Nanostructured lipid carriers co-delivering lapachone and doxorubicin for overcoming multidrug resistance in breast cancer therapy. International journal of nanomedicine 2018, 13, 4107 10.2147/IJN.S163929. 30034236 PMC6047616

[ref11] Guo Y.; He W.; Yang S.; Zhao D.; Li Z.; Luan Y. Co-delivery of docetaxel and verapamil by reduction-sensitive PEG-PLGA-SS-DTX conjugate micelles to reverse the multi-drug resistance of breast cancer. Colloids Surf., B 2017, 151, 119–127. 10.1016/j.colsurfb.2016.12.012. 27988472

[ref12] Xu Y.; Asghar S.; Gao S.; Chen Z.; Huang L.; Yin L.; Ping Q.; Xiao Y. Polysaccharide-based nanoparticles for co-loading mitoxantrone and verapamil to overcome multidrug resistance in breast tumor. Int. J. Nanomed. 2017, 12, 7337 10.2147/IJN.S145620. PMC5644570 29066886

[ref13] Abo Mansour H.; El-Batsh M.; Badawy N.; Mehanna E.; Mesbah N.; Abo-Elmatty D. Effect of co-treatment with doxorubicin and verapamil loaded into chitosan nanoparticles on diethylnitrosamine-induced hepatocellular carcinoma in mice. Human & Experimental Toxicology 2020, 39, 1528–1544. 10.1177/0960327120930266. 32519553

[ref14] Soe Z. C.; Kwon J. B.; Thapa R. K.; Ou W.; Nguyen H. T.; Gautam M.; Oh K. T.; Choi H. G.; Ku S. K.; Yong C. S.; Kim J. O. Transferrin-Conjugated Polymeric Nanoparticle for Receptor-Mediated Delivery of Doxorubicin in Doxorubicin-Resistant Breast Cancer Cells. Pharmaceutics 2019, 11, 63 10.3390/pharmaceutics11020063. 30717256 PMC6410246

[ref15] Qin M.; Lee Y. E. K.; Ray A.; Kopelman R. Overcoming cancer multidrug resistance by codelivery of doxorubicin and verapamil with hydrogel nanoparticles. Macromol. Biosci. 2014, 14, 1106–1115. 10.1002/mabi.201400035. 24771682

[ref16] Ahmadi F.; Bahmyari M.; Akbarizadeh A.; Alipour S. Doxorubicin-verapamil dual loaded PLGA nanoparticles for overcoming P-glycoprotein mediated resistance in cancer: Effect of verapamil concentration. Journal of Drug Delivery Science and Technology 2019, 53, 101206 10.1016/j.jddst.2019.101206.

[ref17] Zhang T.; Ma K.; Huang J.; Wang S.; Liu Y.; Fan G.; Liu M.; Yang G.; Wang C.; Fan P. CDKN2B is critical for verapamil-mediated reversal of doxorubicin resistance in hepatocellular carcinoma. Oncotarget 2017, 8, 110052 10.18632/oncotarget.22123. 29299129 PMC5746364

[ref18] Yusa K.; Tsuruo T. Reversal mechanism of multidrug resistance by verapamil: direct binding of verapamil to P-glycoprotein on specific sites and transport of verapamil outward across the plasma membrane of K562/ADM cells. Cancer Res. 1989, 49, 5002–5006. 2569930

[ref19] Park S. J.; Garcia C. V.; Shin G. H.; Kim J. T. Improvement of curcuminoid bioaccessibility from turmeric by a nanostructured lipid carrier system. Food chemistry 2018, 251, 51–57. 10.1016/j.foodchem.2018.01.071. 29426423

[ref20] Kalaycioglu G. D.; Aydogan N. Preparation and investigation of solid lipid nanoparticles for drug delivery. Colloids and surfaces A: Physicochemical and engineering aspects 2016, 510, 77–86. 10.1016/j.colsurfa.2016.06.034.

[ref21] Zhou S.; Shang Q.; Wang N.; Li Q.; Song A.; Luan Y. Rational design of a minimalist nanoplatform to maximize immunotherapeutic efficacy: Four birds with one stone. J. Controlled Release 2020, 328, 617–630. 10.1016/j.jconrel.2020.09.035. 32976902

[ref22] Vieira R.; Severino P.; Nalone L. A.; Souto S. B.; Silva A. M.; Lucarini M.; Durazzo A.; Santini A.; Souto E. B. Sucupira oil-loaded nanostructured lipid carriers (NLC): Lipid screening, factorial design, release profile, and cytotoxicity. Molecules 2020, 25, 685 10.3390/molecules25030685. 32041134 PMC7038118

[ref23] Haider M.; Abdin S. M.; Kamal L.; Orive G. Nanostructured lipid carriers for delivery of chemotherapeutics: A review. Pharmaceutics 2020, 12, 288 10.3390/pharmaceutics12030288. 32210127 PMC7151211

[ref24] Selvamuthukumar S.; Velmurugan R. Nanostructured lipid carriers: a potential drug carrier for cancer chemotherapy. Lipids Health Dis. 2012, 11, 1–8. 10.1186/1476-511X-11-159. 23167765 PMC3561225

[ref25] Naseri N.; Valizadeh H.; Zakeri-Milani P. Solid lipid nanoparticles and nanostructured lipid carriers: structure, preparation and application. Advanced pharmaceutical bulletin 2015, 5, 305 10.15171/apb.2015.043. 26504751 PMC4616893

[ref26] Müller R.; Radtke M.; Wissing S. Nanostructured lipid matrices for improved microencapsulation of drugs. International journal of pharmaceutics 2002, 242, 121–128. 10.1016/S0378-5173(02)00180-1. 12176234

[ref27] Sun H.; Guo X.; Zeng S.; Wang Y.; Hou J.; Yang D.; Zhou S. A multifunctional liposomal nanoplatform co-delivering hydrophobic and hydrophilic doxorubicin for complete eradication of xenografted tumors. Nanoscale 2019, 11, 17759–17772. 10.1039/C9NR04669K. 31552975

[ref28] Oliveira M. S.; Goulart G. C. A.; Ferreira L. A. M.; Carneiro G. Hydrophobic ion pairing as a strategy to improve drug encapsulation into lipid nanocarriers for the cancer treatment. Expert opinion on drug delivery 2017, 14, 983–995. 10.1080/17425247.2017.1266329. 27892713

[ref29] Mussi S. V.; Sawant R.; Perche F.; Oliveira M. C.; Azevedo R. B.; Ferreira L. A.; Torchilin V. P. Novel nanostructured lipid carrier co-loaded with doxorubicin and docosahexaenoic acid demonstrates enhanced in vitro activity and overcomes drug resistance in MCF-7/Adr cells. Pharm. Res. 2014, 31, 1882–1892. 10.1007/s11095-013-1290-2. 24522814

[ref30] Zhao S.; Minh L. V.; Li N.; Garamus V. M.; Handge U. A.; Liu J.; Zhang R.; Willumeit-Römer R.; Zou A. Doxorubicin hydrochloride-oleic acid conjugate loaded nanostructured lipid carriers for tumor specific drug release. Colloids Surf., B 2016, 145, 95–103. 10.1016/j.colsurfb.2016.04.027. 27137808

[ref31] Zhou T.; Xie S.; Zhou C.; Chen Y.; Li H.; Liu P.; Jiang R.; Hang L.; Jiang G. All-in-one second near-infrared light-responsive drug delivery system for synergistic chemo-photothermal therapy. ACS Applied Bio Materials 2022, 5, 3841–3849. 10.1021/acsabm.2c00208. 35815771

[ref32] Zhang W.; Ding M.; Zhang H.; Shang H.; Zhang A. Tumor acidity and near-infrared light responsive drug delivery MoS2-based nanoparticles for chemo-photothermal therapy. Photodiagnosis and Photodynamic Therapy 2022, 38, 102716 10.1016/j.pdpdt.2022.102716. 35021109

[ref33] Antons J.; Marascio M.; Aeberhard P.; Weissenberger G.; Hirt-Burri N.; Applegate L.; Bourban P.-E.; Pioletti D. Decellularised tissues obtained by a CO2-philic detergent and supercritical CO2. European Cells and Materials 2018, 36, 81–95. 10.22203/eCM.v036a07. 30178445

[ref34] Zhang H.; Gong W.; Wang Z. Y.; Yuan S. J.; Xie X. Y.; Yang Y. F.; Yang Y.; Wang S. S.; Yang D. X.; Xuan Z. X.; Mei X. G. Preparation, Characterization, and Pharmacodynamics of Thermosensitive Liposomes Containing Docetaxel. J. Pharm. Sci. 2014, 103, 2177–2183. 10.1002/jps.24019. 24846075

[ref35] Thakur N. S.; Patel G.; Kushwah V.; Jain S.; Banerjee U. C. Self-assembled gold nanoparticle–lipid nanocomposites for on-demand delivery, tumor accumulation, and combined photothermal–photodynamic therapy. ACS Applied Bio Materials 2019, 2, 349–361. 10.1021/acsabm.8b00618. 35016358

[ref36] Yu N.; Huang L.; Zhou Y.; Xue T.; Chen Z.; Han G. Near-Infrared-Light Activatable Nanoparticles for Deep-Tissue-Penetrating Wireless Optogenetics. Adv. Healthcare Mater. 2019, 8, 1801132 10.1002/adhm.201801132. 30633858

[ref37] Riley R. S.; Day E. S. Gold nanoparticle-mediated photothermal therapy: applications and opportunities for multimodal cancer treatment. Wiley Interdiscip. Rev.: Nanomed. Nanobiotechnol. 2017, 9, e1449 10.1002/wnan.1449. PMC5474189 28160445

[ref38] Altas B. O.; Goktas C.; Topcu G.; Aydogan N. Multi-Stimuli-Responsive Tadpole-like Polymer/Lipid Janus Microrobots for Advanced Smart Material Applications. ACS Appl. Mater. Interfaces 2024, 16, 15533 10.1021/acsami.3c18826. 38356451 PMC10983008

[ref39] Akbarian A.; Ebtekar M.; Pakravan N.; Hassan Z. M. Folate receptor alpha targeted delivery of artemether to breast cancer cells with folate-decorated human serum albumin nanoparticles. Int. J. Biol. Macromol. 2020, 152, 90–101. 10.1016/j.ijbiomac.2020.02.106. 32057865

[ref40] Okuyucu C. E.; Kalaycioglu G. D.; Kacaroglu D.; Ozden A. K.; Aydogan N. Trojan-like doxorubicin and gold nanoparticle entrapped smart nanostructured lipid carriers for breast cancer synergistic chemo/photothermal therapy. Colloids Surf., A 2023, 672, 131763 10.1016/j.colsurfa.2023.131763.

[ref41] Liu M.; Guyot-Sionnest P. Mechanism of silver (I)-assisted growth of gold nanorods and bipyramids. J. Phys. Chem. B 2005, 109, 22192–22200. 10.1021/jp054808n. 16853888

[ref42] Nikoobakht B.; El-Sayed M. A. Preparation and growth mechanism of gold nanorods (NRs) using seed-mediated growth method. Chemistry of materials 2003, 15, 1957–1962. 10.1021/cm020732l.

[ref43] Han F.; Li S.; Yin R.; Liu H.; Xu L. Effect of surfactants on the formation and characterization of a new type of colloidal drug delivery system: nanostructured lipid carriers. Colloids Surf., A 2008, 315, 210–216. 10.1016/j.colsurfa.2007.08.005.

[ref44] Joshi M.; Pathak S.; Sharma S.; Patravale V. Design and in vivo pharmacodynamic evaluation of nanostructured lipid carriers for parenteral delivery of artemether: Nanoject. International journal of pharmaceutics 2008, 364, 119–126. 10.1016/j.ijpharm.2008.07.032. 18765274

[ref45] Garcia M.; Platet N.; Liaudet E.; Laurent V.; Derocq D.; Brouillet J.-P.; Rochefort H. Biological and clinical significance of cathepsin D in breast cancer metastasis. Stem cells 1996, 14, 642–650. 10.1002/stem.140642. 8948022

[ref46] Hu F.-Q.; Jiang S.-P.; Du Y.-Z.; Yuan H.; Ye Y.-Q.; Zeng S. Preparation and characterization of stearic acid nanostructured lipid carriers by solvent diffusion method in an aqueous system. Colloids Surf., B 2005, 45, 167–173. 10.1016/j.colsurfb.2005.08.005. 16198092

[ref47] Wairkar S.; Patel D.; Singh A. Nanostructured lipid carrier based dermal gel of cyclosporine for atopic dermatitis-in vitro and in vivo evaluation. Journal of Drug Delivery Science and Technology 2022, 72, 103365 10.1016/j.jddst.2022.103365.

[ref48] Mackey M. A.; Ali M. R.; Austin L. A.; Near R. D.; El-Sayed M. A. The most effective gold nanorod size for plasmonic photothermal therapy: theory and in vitro experiments. J. Phys. Chem. B 2014, 118, 1319–1326. 10.1021/jp409298f. 24433049 PMC3983380

[ref49] Near R. D.; Hayden S. C.; El-Sayed M. A. Thin to thick, short to long: spectral properties of gold nanorods by theoretical modeling. J. Phys. Chem. C 2013, 117, 18653–18656. 10.1021/jp4078344.

[ref50] Zheng S.; Nguyen V. D.; Song S. Y.; Han J.; Park J.-O. Combined photothermal-chemotherapy of breast cancer by near infrared light responsive hyaluronic acid-decorated nanostructured lipid carriers. Nanotechnology 2017, 28, 435102 10.1088/1361-6528/aa847f. 28783035

[ref51] Zhao J.; Wang A.; Si T.; Hong J.-D.; Li J. Gold nanorods based multicompartment mesoporous silica composites as bioagents for highly efficient photothermal therapy. J. Colloid Interface Sci. 2019, 549, 9–15. 10.1016/j.jcis.2019.04.051. 31015057

[ref52] Huang X.; Jain P. K.; El-Sayed I. H.; El-Sayed M. A. Plasmonic photothermal therapy (PPTT) using gold nanoparticles. Lasers in medical science 2008, 23, 217–228. 10.1007/s10103-007-0470-x. 17674122

[ref53] Abdolahpour S.; Mahdieh N.; Jamali Z.; Akbarzadeh A.; Toliyat T.; Paknejad M. Development of doxorubicin-loaded nanostructured lipid carriers: preparation, characterization, and in vitro evaluation on MCF-7 cell line. BioNanoScience 2017, 7, 32–39. 10.1007/s12668-016-0391-x.

[ref54] Zhou J.; Chen S.; Sun C.; Du Q.; Luo P.; Du B.; Yao H. A “submunition” dual-drug system based on smart hollow NaYF 4/apoferritin nanocage for upconversion imaging. RSC Adv. 2016, 6, 33443–33454. 10.1039/C5RA24285A.

[ref55] Kalaria D. R.; Sharma G.; Beniwal V.; Ravi Kumar M. N. V. Design of Biodegradable Nanoparticles for Oral Delivery of Doxorubicin: In vivo Pharmacokinetics and Toxicity Studies in Rats. Pharm. Res. 2009, 26, 492–501. 10.1007/s11095-008-9763-4. 18998202

[ref56] Yoshida M. I.; Gomes E. C. L.; Soares C. D. V.; Cunha A. F.; Oliveira M. A. Thermal analysis applied to verapamil hydrochloride characterization in pharmaceutical formulations. Molecules 2010, 15, 2439–2452. 10.3390/molecules15042439. 20428054 PMC6257305

[ref57] Lages E. B.; Fernandes R. S.; de Oliveira Silva J.; de Souza Â. M.; Cassali G. D.; de Barros A. L. B.; Ferreira L. A. M. Co-delivery of doxorubicin, docosahexaenoic acid, and α-tocopherol succinate by nanostructured lipid carriers has a synergistic effect to enhance antitumor activity and reduce toxicity. Biomed. Pharmacother. 2020, 132, 110876 10.1016/j.biopha.2020.110876. 33113428

[ref58] Zara G. P.; Cavalli R.; FUNDARÒ A.; Bargoni A.; CAPUTO O.; GASCO M. R. Pharmacokinetics of doxorubicin incorporated in solid lipid nanospheres (SLN). Pharmacological research 1999, 40, 281–286. 10.1006/phrs.1999.0509. 10479474

[ref59] Zhang T.; Zheng Y.; Peng Q.; Cao X.; Gong T.; Zhang Z. A novel submicron emulsion system loaded with vincristine–oleic acid ion-pair complex with improved anticancer effect: in vitro and in vivo studies. International journal of nanomedicine 2013, 1185–1196. 10.2147/IJN.S41775. 23658485 PMC3607420

[ref60] Jusu S. M.; Obayemi J. D.; Salifu A. A.; Nwazojie C. C.; Uzonwanne V. O.; Odusanya O. S.; Soboyejo W. O. Plga-cs-peg microparticles for controlled drug delivery in the treatment of triple negative breast cancer cells. Applied Sciences 2021, 11, 7112 10.3390/app11157112.

[ref61] Sathiyaseelan A.; Saravanakumar K.; Manivasagan P.; Jeong M. S.; Jang E.-S.; Wang M.-H. Folic acid conjugated chitosan encapsulated palladium nanoclusters for NIR triggered photothermal breast cancer treatment. Carbohydr. Polym. 2022, 280, 119021 10.1016/j.carbpol.2021.119021. 35027124

[ref62] Liu Y.; Zhang X.; Luo L.; Li L.; Zhu R. Y.; Li A.; He Y.; Cao W.; Niu K.; Liu H.; Yang J.; Gao D. Gold-nanobranched-shell based drug vehicles with ultrahigh photothermal efficiency for chemo-photothermal therapy. Nanomed.: Nanotechnol., Biol. Med. 2019, 18, 303–314. 10.1016/j.nano.2018.09.015. 30326275

[ref63] Liu Y.; Zhang X.; Liu Z.; Wang L.; Luo L.; Wang M.; Wang Q.; Gao D. Gold nanoshell-based betulinic acid liposomes for synergistic chemo-photothermal therapy. Nanomedicine: Nanotechnology, Biology and Medicine 2017, 13, 1891–1900. 10.1016/j.nano.2017.03.012. 28363771

[ref64] Rezaei N.; Zarkesh I.; Fotouhi A.; Alikhani H. K.; Hassan M.; Vosough M. Chitosan-coated nanoparticles in innovative cancer bio-medicine. Drug Dev. Res. 2024, 85, e22189 10.1002/ddr.22189. 38678548

[ref65] Fai A. E. C.; Stamford T. C. M.; Stamford-Arnaud T. M.; Santa-Cruz P. D.; da Silva M. C. F.; Campos-Takaki G. M.; Stamford T. L. M. Physico-chemical characteristics and functional properties of chitin and chitosan produced by Mucor circinelloides using yam bean as substrate. Molecules 2011, 16, 7143–7154. 10.3390/molecules16087143. 21862956 PMC6264275

[ref66] Hasegawa M.; Yagi K.; Iwakawa S.; Hirai M. Chitosan induces apoptosis via caspase-3 activation in bladder tumor cells. Japanese journal of cancer research 2001, 92, 459–466. 10.1111/j.1349-7006.2001.tb01116.x. 11346469 PMC5926722

[ref67] Alomrani A.; Badran M.; Harisa G. I.; ALshehry M.; Alhariri M.; Alshamsan A.; Alkholief M. The use of chitosan-coated flexible liposomes as a remarkable carrier to enhance the antitumor efficacy of 5-fluorouracil against colorectal cancer. Saudi pharmaceutical journal 2019, 27, 603–611. 10.1016/j.jsps.2019.02.008. 31297013 PMC6598218

[ref68] Shen Z.; Li Y.; Kohama K.; Oneill B.; Bi J. Improved drug targeting of cancer cells by utilizing actively targetable folic acid-conjugated albumin nanospheres. Pharmacol. Res. 2011, 63, 51–58. 10.1016/j.phrs.2010.10.012. 21035550

[ref69] Ahmadi M.; Ritter C. A.; von Woedtke T.; Bekeschus S.; Wende K. Package delivered: folate receptor-mediated transporters in cancer therapy and diagnosis. Chemical Science 2024, 15, 1966–2006. 10.1039/D3SC05539F. 38332833 PMC10848714

[ref70] Chen C.; Ke J.; Zhou X. E.; Yi W.; Brunzelle J. S.; Li J.; Yong E.-L.; Xu H. E.; Melcher K. Structural basis for molecular recognition of folic acid by folate receptors. Nature 2013, 500, 486–489. 10.1038/nature12327. 23851396 PMC5797940

