# Supplementary material for: Chemosensitizer Loaded NIR-Responsive Nanostructured Lipid Carriers: A Tool for Drug-Resistant Breast Cancer Synergistic Therapy
Source: ACS Appl Bio Mater. 2025 Feb 18;8(3):2167–81. doi: 10.1021/acsabm.4c01675 (PMC11921034; doi:10.1021/acsabm.4c01675)
Supplement: Supplementary file 1 — mt4c01675_si_001.pdf [file mt4c01675_si_001.pdf]

# CHEMOSENSITIZER LOADED NIR-RESPONSIVE NANOSTRUCTURED LIPID CARRIERS: A TOOL FOR DRUG-RESISTANT BREAST CANCER SYNERGISTIC THERAPY

**Cigdemnaz Ersoz Okuyucu[1], Gokce Dicle Kalaycioglu[1], Ayse Kevser Ozden[2] and Nihal Aydogan[1]***

**[1]Hacettepe University, Department of Chemical Engineering, Beytepe 06800, Ankara, Turkey**

**[2]Lokman Hekim University, Faculty of Medicine, Medical Biology Department, 06530, Ankara, Turkey**

**Table S1.** Size changes with an increasing amount of oleic acid.

| | | Size (nm) | |
|---|---|---|---|
| **OA Content (%)** | **PDI** | **DLS** | **AFM** |
| 20 | 0.38 ± 0.06 | 105.84 ± 40.57 | 135.20 ± 48.48 |
| 30 | 0.12 ± 0.06 | 72.02 ± 31.39 | 66.35 ± 14.85 |
| 40 | 0.16 ± 0.06 | 205.40 ± 79.64 | 109.02 ± 32.19 |

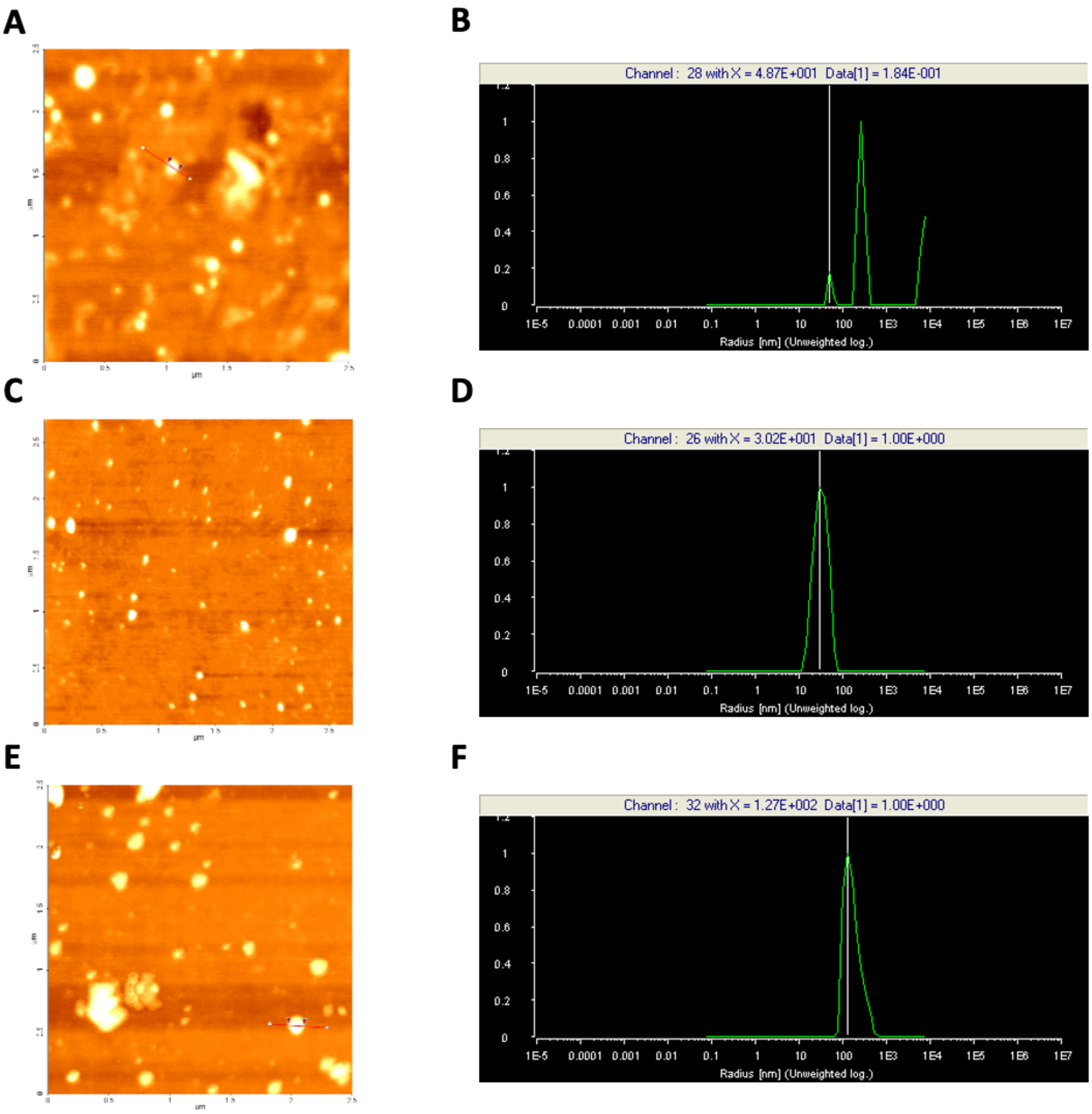


**Figure S1.** A) AFM images of 20% OA containing NLC, B) Particle size distribution of 20% OA containing NLC determined by DLS, C) AFM images of 30% OA containing NLC, D) Particle size distribution of 30% OA containing NLC determined by DLS, E) AFM images of 40% OA containing NLC, F) Particle size distribution of 40% OA containing NLC determined by DLS (Topography mode was used for the AFM images).

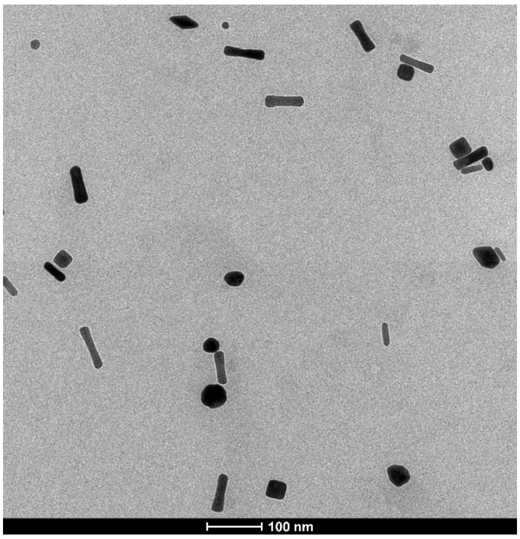


**Figure S2**. Bright field TEM image of  AuNRs (The bar is 100 nm for the image).

**Table S2.** Size changes NLC with an increasing concentration of AuNRs .

| | Concentration (µg Au/mg lipid) | DLS (nm) | PDI | AFM (nm) |
|---|---|---|---|---|
| **AuNRs NLC** | **19.5** | 227.76 ± 81.78 | 0.507 ± 0.02 | 302.13 ± 62.98 |
| | **26** | 253.37 ± 43.35 | 0.483 ± 0.03 | 382.04 ± 81.21 |
| | **52** | 302.41 ± 83.47 | 0.483 ± 0.03 | 326.63 ± 73.51 |

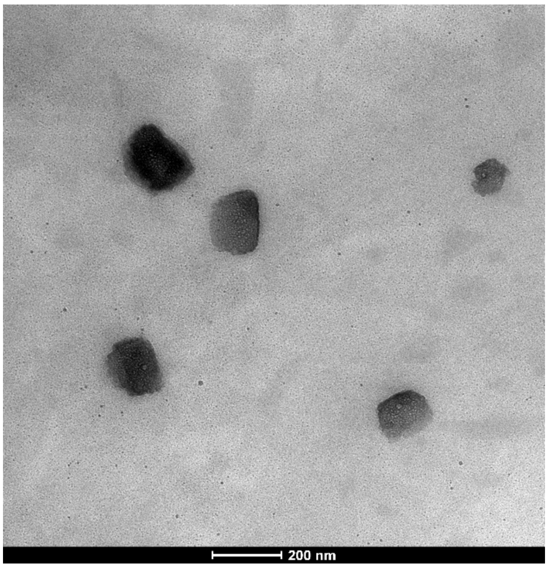


**Figure S3.** Bright field TEM image of AuNRs NLC at 19.5 µg Au/mg lipid concentration (The bar is 200 nm for the image).

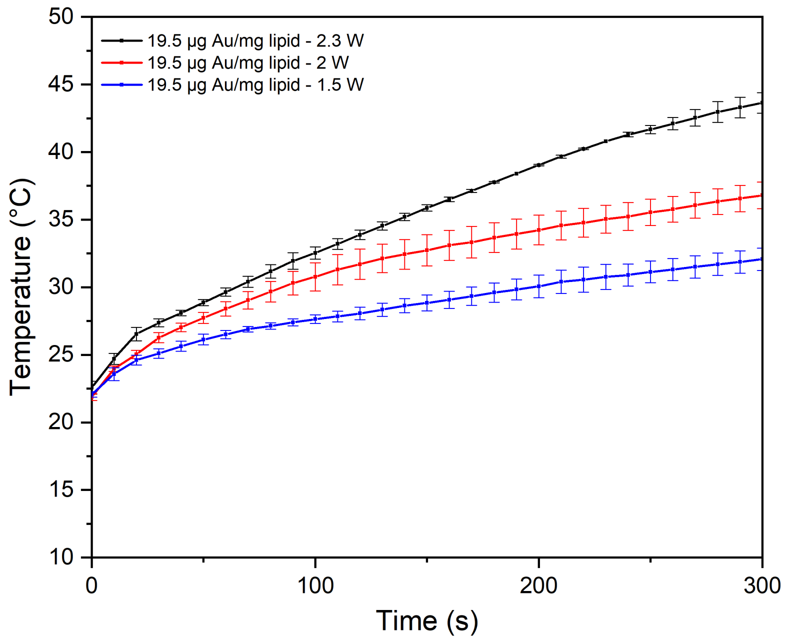


**Figure S4**. Temperature change profiles of different power (1.5-2.3 W) applied NLC dispersions at a concentration of 19.5 µg Au/mg lipid. Data were presented as mean ± SD (n=3).

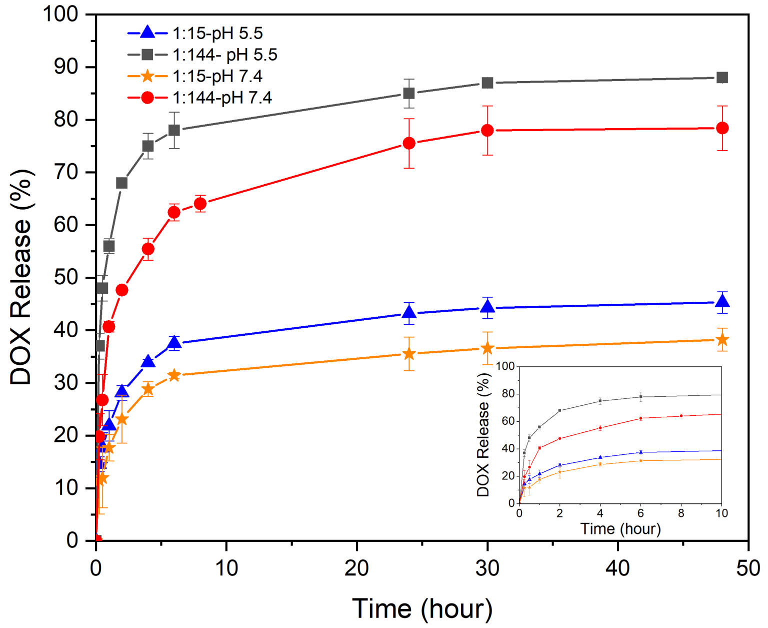


**Figure S5.** In vitro release profile of DOX from DOX NLC in the ratio of 1:144 and 1:15 (drug:lipid) at pH 7.4 and pH 5.5 in PBS solution (including 0.7% Tween-20 (w/v%)) during 48 hours at 37 °C. Inset shows both DOX and DOX-OA release over a shorter period of time (10 h). Data were presented as mean ± SD (n=3).

**Table S3.** Melting temperature (Tm (°C)), enthalpy (ΔH (J/g)) and crystallinity index (CI%) of DOX NLC, DOX-OA NLC, VERA NLC, DOX-OA+VERA NLC.

| **Sample** | NLC | | DOX NLC | | DOX-OA NLC | | VERA NLC | | DOX-OA +VERA NLC | |
|---|---|---|---|---|---|---|---|---|---|---|
| **$T_m$ (°C)** | 43.6 | 57.1 | 48.0 | 59.8 | 47.5 | 58.4 | 47.7 | 59.5 | 47.0 | 57.7 |
| **ΔH (J/g)** | 29.7 | | 30.6 | | 24.8 | | 21.1 | | 20.8 | |
| **CI %** | 13.9 | | 14.3 | | 11.5 | | 9.8 | | 9.7 | |

**Table S4.** Melting temperature (Tm (°C)), enthalpy (ΔH (J/g)) and crystallinity index (CI%) of AuNRs NLC, DOX+AuNRs NLC, DOX-OA+AuNRs NLC, VERA+AuNRs NLC and (DOX-OA+VERA+AuNRs)@NLC.

| **Sample** | AuNRs NLC | | DOX+AuNRs NLC | | DOX-OA+AuNRs NLC | | VERA+AuNRs NLC | | (DOX-OA+ VERA+ AuNRs) @NLC | |
|---|---|---|---|---|---|---|---|---|---|---|
| **$T_m$ (°C)** | 27.91 | 59.42 | 49.9 | 62.23 | 48.88 | 61.88 | 51.58 | 60.70 | 48.90 | 61.07 |
| **ΔH (J/g)** | 9.04 | | 2.92 | | 6.20 | | 6.70 | | 6.54 | |
| **CI %** | 4.22 | | 1.36 | | 2.90 | | 3.13 | | 3.05 | |

A B

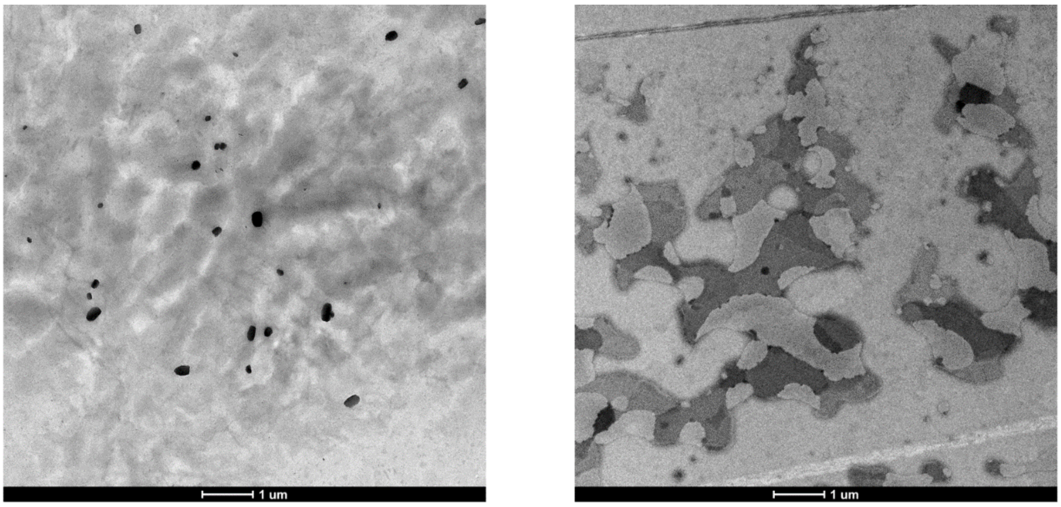


**Figure S6.** A) Bright field TEM image of AuNRs NLC without NIR laser irradiation and B) Bright field TEM image of (DOX-OA+VERA+AuNRs)@NLC after 5 cycle NIR laser irradiation. (The bar is 1 μm for TEM images).

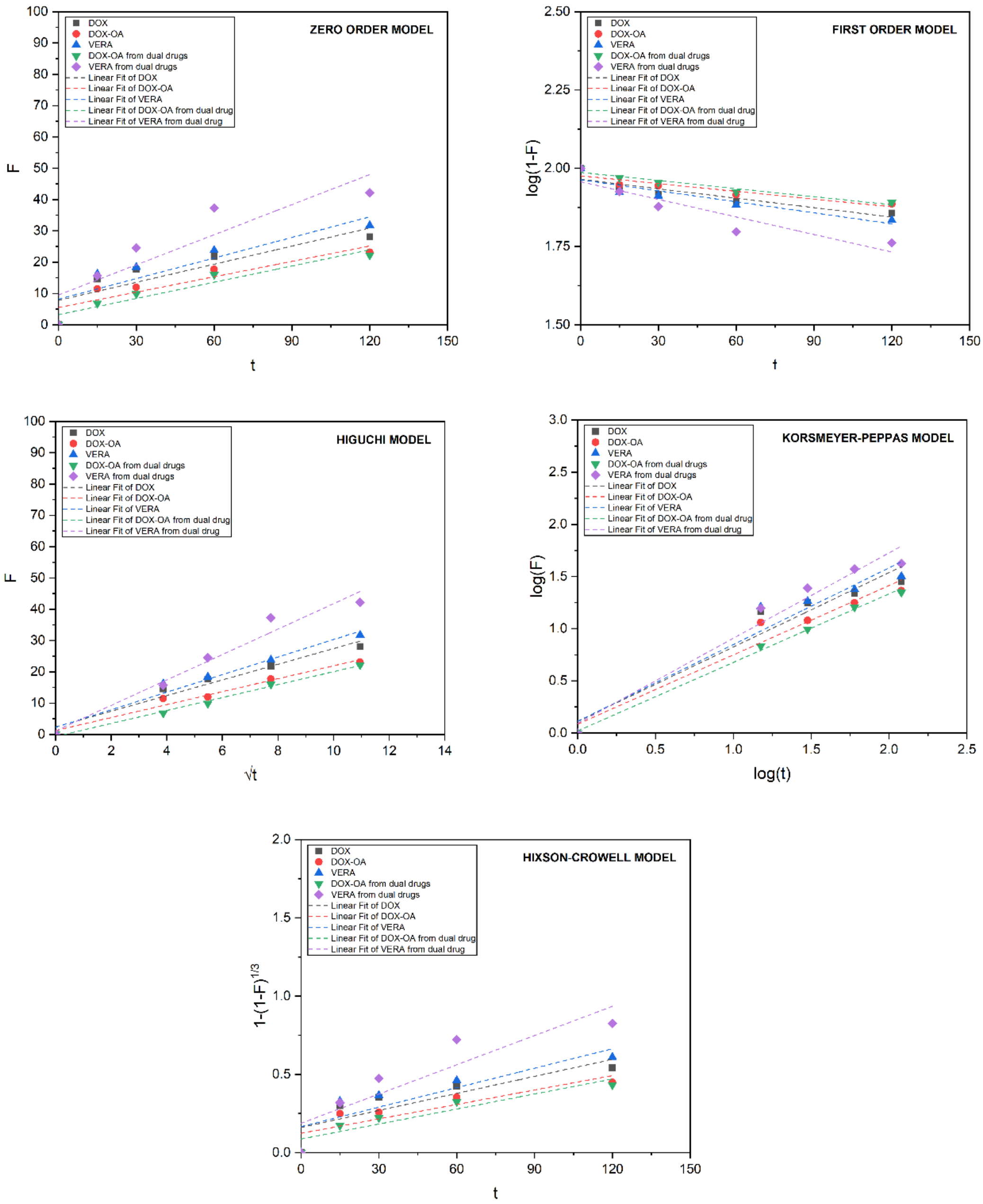


**Figure S7.** Release kinetic model profiles of DOX, DOX-OA, VERA from NLC and DOX-OA and VERA from dual drug-loaded NLC in the ratio of 1:15 (drug:lipid) at pH 5.5.

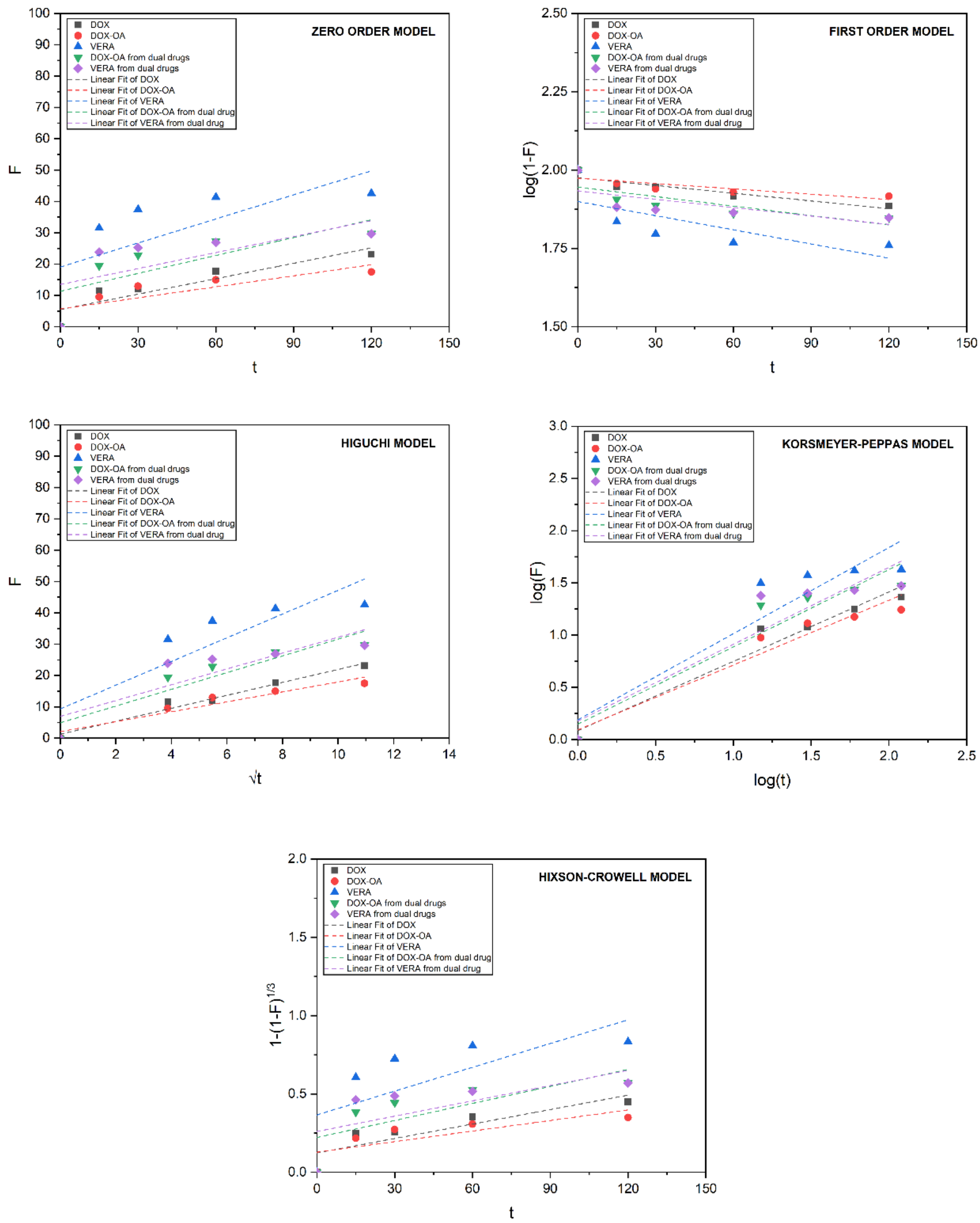


**Figure S8.** Release kinetic model profiles of DOX, DOX-OA, VERA from NLC and DOX-OA and VERA from dual drug-loaded NLC in the ratio of 1:15 (drug:lipid) at pH 7.4.

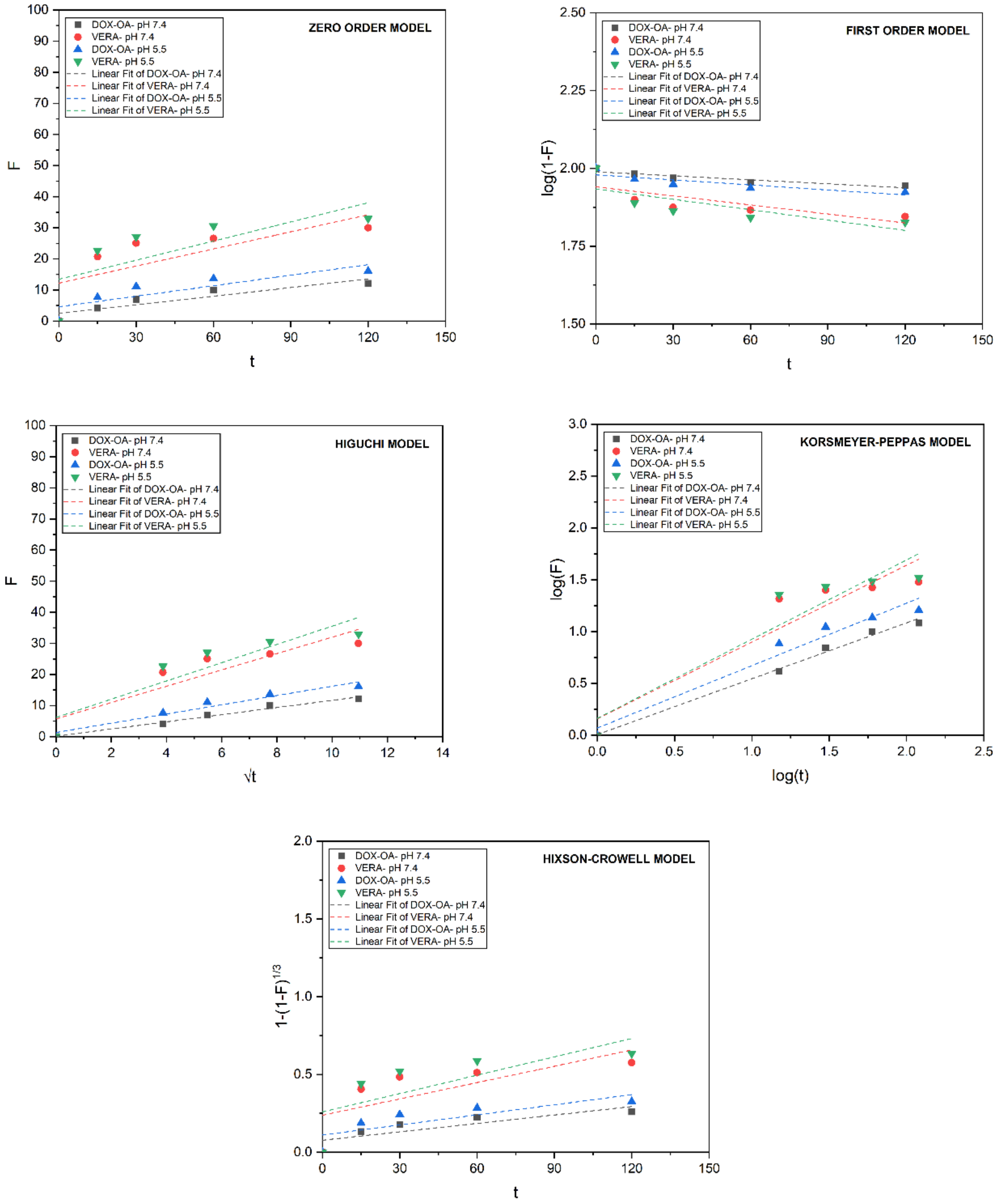


**Figure S9.** Release kinetic model profiles of DOX-OA and VERA from (DOX-OA+VERA+AuNRs)@NLC in the ratio of 1:15 (drug:lipid) at pH 7.4 and pH 5.5.

**Table S5.** IC50 of the formulations with 808 nm NIR laser irradiation at 2.3 W at 48 h.

| Formulation | IC50 (ug/ml) | |
|---|---|---|
| | MDA-MB-231[R] | MDA-MB-231 |
| DOX-OA NLC | 4.6 ±0.22 | 3.8 ±0.12 |
| (DOX-OA+VERA)@NLC | 2.3 ±0.10 | 2.0 ±0.10 |
| (DOX-OA+VERA+AuNRs)@NLC | 1.5 ±0.05 | 2.1 ±0.10 |
| DOX-OA+VERA | 7.8 ±0.33 | 6.8 ±0.32 |
| DOX-OA | 13.6 ±0.59 | 6.7 ±0.31 |
